# Efficacy of Dietary Therapy for Eosinophilic Esophagitis in Children and Adults: An Updated Systematic Review and Meta-Analysis

**DOI:** 10.3390/nu16142231

**Published:** 2024-07-11

**Authors:** Ángel Arias, Antonio Tejera-Muñoz, Lucía Gutiérrez-Ramírez, Javier Molina-Infante, Alfredo J. Lucendo

**Affiliations:** 1 Research Unit Complejo Hospitalario La Mancha Centro, 13600 Alcázar de San Juan, Spain; aariasa@sescam.jccm.es (Á.A.); atejeram@sescam.jccm.es (A.T.-M.); lgutierrezramirez@sescam.jccm.es (L.G.-R.); 2 Instituto de Investigación Sanitaria de Castilla-La Mancha (IDISCAM), 45004 Toledo, Spain; 3 Centro de Investigación Biomédica en Red Enfermedades Hepáticas y Digestivas (CIBEREHD), 28006 Madrid, Spain; xavi_molina@hotmail.com; 4 Instituto de Investigación Sanitaria Princesa, 20006 Madrid, Spain; 5 Fundación del Hospital Nacional de Parapléjicos para la Investigación y la Integración, 45007 Toledo, Spain; 6 Department of Gastroenterology, Hospital Universitario San Pedro de Alcantara, 10003 Caceres, Spain; 7 Department of Gastroenterology, Hospital General de Tomelloso, Tomelloso, 13700 Ciudad Real, Spain

**Keywords:** eosinophilic esophagitis, dietary treatment, histological remission, pediatric, cohort studies, meta-analysis

## Abstract

Background: Several dietary approaches have been used to induce remission in patients with eosinophilic esophagitis (EoE), yielding varied results. Methods: We searched the MEDLINE, EMBASE, and Scopus databases up to May 2024 to identify studies including dietary interventions for EoE used as monotherapy. Summary estimates with 95% CIs for achieving <15 eosinophils/HPF were calculated for each approach. Fixed or random effects models were used depending on heterogeneity (I^2^); publication bias risks were assessed using funnel plot analyses. Subgroup analyses results were compared using meta-regression. Results: Forty-three studies with 2825 patients were included in quantitative summaries. The overall rate of histologic remission was 60.6% (95% CI, 54.6–66.5%). Effectiveness rates were 94.5% (95% CI, 92.3–96.4%) for elemental diets, 63.9% (95% CI, 58.5–69.2%) for six-food elimination diets, 54.7% (95% CI, 45.7–63.6%) for four-food elimination diets, 44.3% (95% CI, 36.1–52.8%) for two-food elimination diets, 46.4% (95% CI, 40–52.9%) for one-food elimination diets, and 39.5% (95% CI, 30.3–49.2%) for allergy testing-directed food elimination diets. Overall, superior efficacy was noted in children than in adults and in retrospective compared to prospective studies. Conclusion: Diet therapy remains an effective therapeutic asset for pediatric and adult patients with EoE, with increasing efficacy noted as the levels of dietary restriction increase.

## 1. Introduction

Eosinophilic esophagitis (EoE) has emerged in recent decades as an esophageal immune-mediated disease, mainly affecting children and young adults in the late phase of the so-called atopic march [1,2]. EoE is predominantly triggered by food antigens, so dietary therapy is the only treatment specifically targeting the cause of the disease [1]. There are three main dietary-based approaches for EoE therapy: elemental diet (oral feeding based exclusively on amino acid-based formulas), food elimination based on food allergy testing, and empiric elimination diet (eliminating those food groups know to be the most common to trigger EoE locally) [3]. The first meta-analysis on dietary therapy published by our group in 2014 showed that the most restrictive diets (elemental diet and empiric six-food elimination diet (SFED)) were the most effective schemes, with histological remission rates of 90% and 72%, respectively [4]. Conversely, a food allergy testing-based elimination diet was demonstrated to be a less effective approach (mean efficacy 45%), especially in adults [4]. At that time, studies on an empiric four-food elimination diet (FFED) and other easier optimized dietary schemes had not been published yet.

Over the past decade, a major breakthrough has been the simplification of empiric dietary restrictions, along with the implementation of a more rational step-up approach for dietary therapy in EoE. The rationale for a FFED was that all SFED studies evaluating individual food reintroduction in responders revealed that nuts and fish/seafood were almost negligible triggers for EoE in both children and adults [5,6,7]. The first studies on an empiric FFED showed high efficacy for adults (54%) [8] and children (64%) [9], with a majority of patients showing just one or two food triggers after food reintroduction, so there was clearly room for improvement. The seminal study first using a step-up approach (2-4-6) [3] was instrumental to understand that an increasing level of food restriction could avoid unnecessary food restrictions, save endoscopic procedures, and shorten the diagnostic process. As a matter of fact, a recent study based on a theoretical computational model proved that that the step-up approach, always starting with elimination of dairy, is the most efficient strategy in dietary therapy [10]. More recently, the first study on a milk-elimination diet in children disclosed a 50% efficacy [11].

Controversial results that contrast with previous findings were published last year. The first randomized trial on dietary therapy reported somewhat counterintuitive results since a milk or one-food elimination diet (OFED) showed a similar efficacy compared to a SFED (34% vs. 40%, *p* = 0.58) [12]. Taking into account major advances in this field over the past decade, the aim of the present study is to conduct a systematic review and meta-analysis on dietary therapy for EoE in order to update our previous data from 2014 and shed some light on the controversial results recently reported.

## 2. Materials and Methods

### 2.1. Study Protocol

The protocol was registered on PROSPERO (CRD42024495950); the study was reported in accordance with the Preferred Reporting for Systematic Reviews and Meta-Analysis (PRISMA) guidelines [13].

### 2.2. Selection of Studies and Search Strategy

A systematic literature search was performed independently by two authors (AJL and AA) using three major bibliographical databases (PubMed, EMBASE, and Scopus) from interception until December 2023. An updated search for new documents was performed in May 2024. The search was not restricted with regard to date or language of publication, study design, or type of report (i.e., full paper or conference abstract). Individual case reports were excluded.

To retrieve all published reports describing the effectiveness of dietary interventions to induce remission in patients with EoE, we consulted the thesauri for MEDLINE (MESH) and EMBASE (EMTREE) using the following search strategy: “Eosinophilic Esophagitis” [MeSH] OR “Eosinophilic oesophagitis” [MeSH] AND (diet OR dieta* OR diete*). For the Scopus database, only free text searches with truncations were carried out (Table S1). The search was limited to titles and abstracts. To identify additional relevant articles, a hand search of the reference lists of the related documents was performed. Three reviewers (AA, LG-R, and AT-M) independently screened the database search for titles and abstracts. If any of the reviewers considered a title or an abstract might meet the study eligibility criteria, the full text of the study was retrieved.

### 2.3. Inclusion and Exclusion Criteria

Eligibility criteria for studies were to report the effectiveness of any dietary intervention, used as a monotherapy, to induce remission in patients of all ages with confirmed EoE, according to current clinical and histological criteria [1,14]. Patients concomitantly treated with corticosteroids or biologic drugs were excluded, whereas those co-treated with proton pump inhibitor (PPI) therapy were included when nonresponse to PPIs was previously demonstrated based on esophageal biopsies. 

Dietary interventions were defined as any treatment modality consisting of food avoidance from patients’ diets, including elemental diet, elimination diet guided by either blood or skin food allergy testing, and empirical elimination diet. Adherence to the pre-defined protocol or each source study was assessed, and those patients or studies in which patients were managed differently were excluded. For studies assessing the effectiveness of dietary interventions in which at least a subset of included patients met inclusion criteria for this review, these data were extracted even when other data could not be used (i.e., studies assessing effectiveness of several treatment modalities, one of them being a diet-based modality). 

Exclusion criteria for studies included guidelines, reviews, individual case reports, editorials, and letters not providing original information on a dietary-based intervention to treat EoE. Duplicate papers, laboratory studies evaluating the impact of dietary therapy on esophageal cells, and studies using cohorts from previous papers by the same research group were also excluded. Authors were contacted for further clarification when required.

### 2.4. Data Extraction

Three reviewers (AA, LG-R, and AT-M) independently extracted relevant information from each eligible study using a standardized data extraction sheet. Results were cross-checked and discrepancies were solved by consensus. Extracted data included last name of the first author, publication year, study location (country), study period, study design, population by age (children, adolescents, adults, multiple), sample size, number of subjects by sex (if available), type of dietary therapy (and number of patients per modality if several were evaluated), and treatment length, whenever available. Efficacy data included histological response and clinical response rates. EoE remission was considered as presenting less than 15 eosinophils/HPF in esophageal biopsies after dietary intervention. Clinical response, which is heterogeneously collected in clinical studies, was evaluated as defined by authors. Disagreements between reviewers regarding data extraction were clarified through discussion or consulting a senior author (AA and AJL).

### 2.5. Risk of Bias Assessment

Retrieved documents were duplicate reviewed (AJL and AA) for risk of bias using the Cochrane’s Robins-I (Risk of Bias in Non-Randomized Studies—of Intervention) [15] or RoB2 [16] tools, according to the study design. A study was considered to be at low risk of bias if all bias items could be categorized as low risk, whilst studies showing a high risk of bias were those in which any of the items was considered to be high risk.

### 2.6. Study Outcomes and Statistical Analysis

Summary estimates, along with their 95% confidence intervals (CIs), were calculated for the efficacy of each dietary intervention in the per-protocol population with fixed or random effects meta-analyses weighted for the inverse variance following DerSimonian and Laird’s Method [17]. 

Inconsistency between studies was assessed by means of a chi-square test (Cochran Q statistic) and quantified with the I^2^ statistic. Generally, I^2^ was used to evaluate the level of heterogeneity, assigning the categories low, moderate, and high to I^2^ values of 25%, 50%, and 75%, respectively [18]. Publication bias was evaluated with the aid of a funnel plot, and asymmetry was calculated using Begg–Mazumda’s rank test [19] or Egger’s test [20]. Meta-analyses were performed with StatsDirect statistical software version 2.7.9 (StatsDirect Ltd., Cheshire, UK).

### 2.7. Subgroup Analysis

Planned subgroup analyses of the primary outcomes were performed based on different types of dietary intervention, patient age group, document type (full paper or abstract), study design (prospective, retrospective, randomized controlled trial), geographical origin of the study, and study time.

In order to appraise how study methods or extracted data could influence results obtained in the meta-analysis, subgroup analyses were planned according to type of publication (full paper or abstract), patients’ age (children/adolescents versus adults), geographical origin of the data, and risk of bias in source documents. Subgroup differences in estimates were calculated with the aid of random effects meta-regression using aggregate-level data. These analyses were carried out with Stata 13.0 (Statacorp, College Station, TX, USA).

### 2.8. Certainty of Evidence

The certainty of the evidence for the primary outcomes evaluated in the meta-analysis were judged using the GRADE approach [21]. This specifies the certainty for a body of evidence for each outcome as high, moderate, low, and very low by considering five domains: risk of bias, inconsistency, indirectness, imprecision, and publication bias [22].

## 3. Results

### 3.1. Literature Search Results and Characteristics of Included Documents

**Figure 1:**
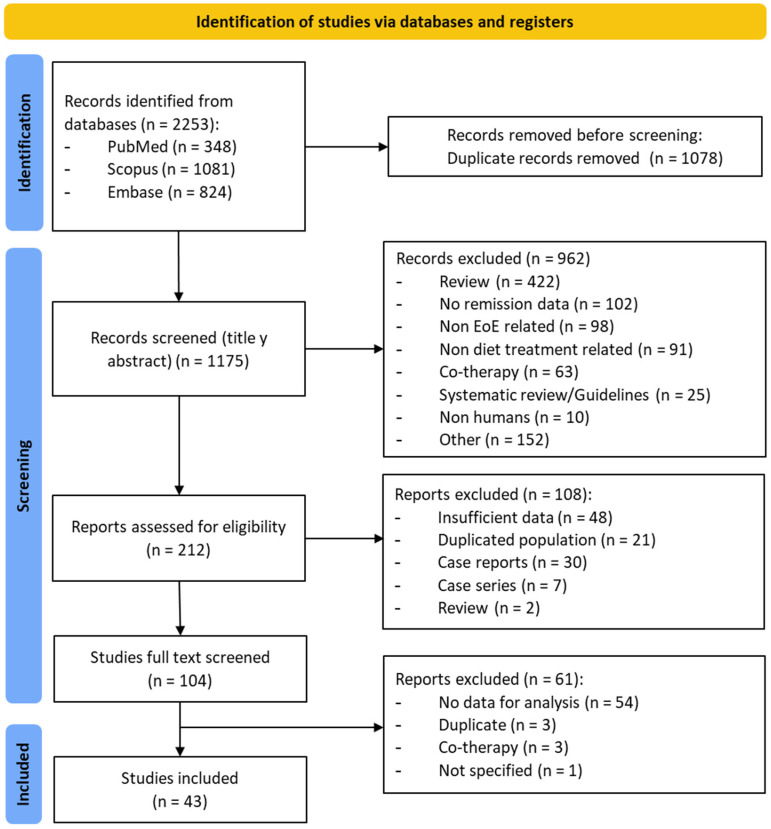
PRISMA flow diagram of study selection process.

Overall, our search strategy retrieved a total of 2252 documents, with 1174 remaining documents once duplicates were removed. After title and abstract examination, 962 documents were excluded since they did not meet inclusion criteria. This yielded 212 potentially relevant documents (Figure 1), of which 43 documents, including 38 full papers [6,7,8,9,11,12,23,24,25,26,27,28,29,30,31,32,33,34,35,36,37,38,39,40,41,42,43,44,45,46,47,48,49,50,51,52,53,54] and 5 abstracts [55,56,57,58,59], were eventually included. These studies combined 2825 EoE patients from 15 different countries. Among them, 136 did not complete the dietary protocol and were excluded from per protocol meta-analyses. As for the study design, 21 were prospective observational studies [6,7,8,9,11,23,26,27,30,33,35,39,40,42,44,45,46,53,55,57,58], 19 were retrospective [24,25,28,29,31,32,34,36,37,38,41,43,47,48,49,51,52,54,56], and 3 were randomized controlled trials [12,50,59]. The sample size of EoE cohorts varied between 4 and 470 patients.

All documents were published between 1995 and 2023. Overall, 27 studies were conducted in North America, including USA [6,9,11,12,23,24,25,28,29,31,33,36,38,41,43,45,46,48,49,51,52,53,55,56,58,59] and Canada [47], whereas 13 studies were carried out in Europe, including Spain [7,8,27,30,35,42], The Netherlands [39,44,50], Italy [26,54], Slovenia [37], and France [34]. An Italian study included a cohort of patients from the United Kingdom [54]. Three additional papers were published in Australia [40,57] and Saudi Arabia, respectively [32].

Studies reported information on 2825 patients overall, comprising 1389 in the pediatric age group (<18 year) and 1104 adults. Age was not defined for the remaining 332 patients. Details from included studies, including sample size and type of dietary approach assessed, are shown in Table 1. Excluded studies after full-text review, along with reasons for exclusion, are displayed in Table S2.

### 3.2. Risk of Bias and Quality Assessment

**Figure 2:**
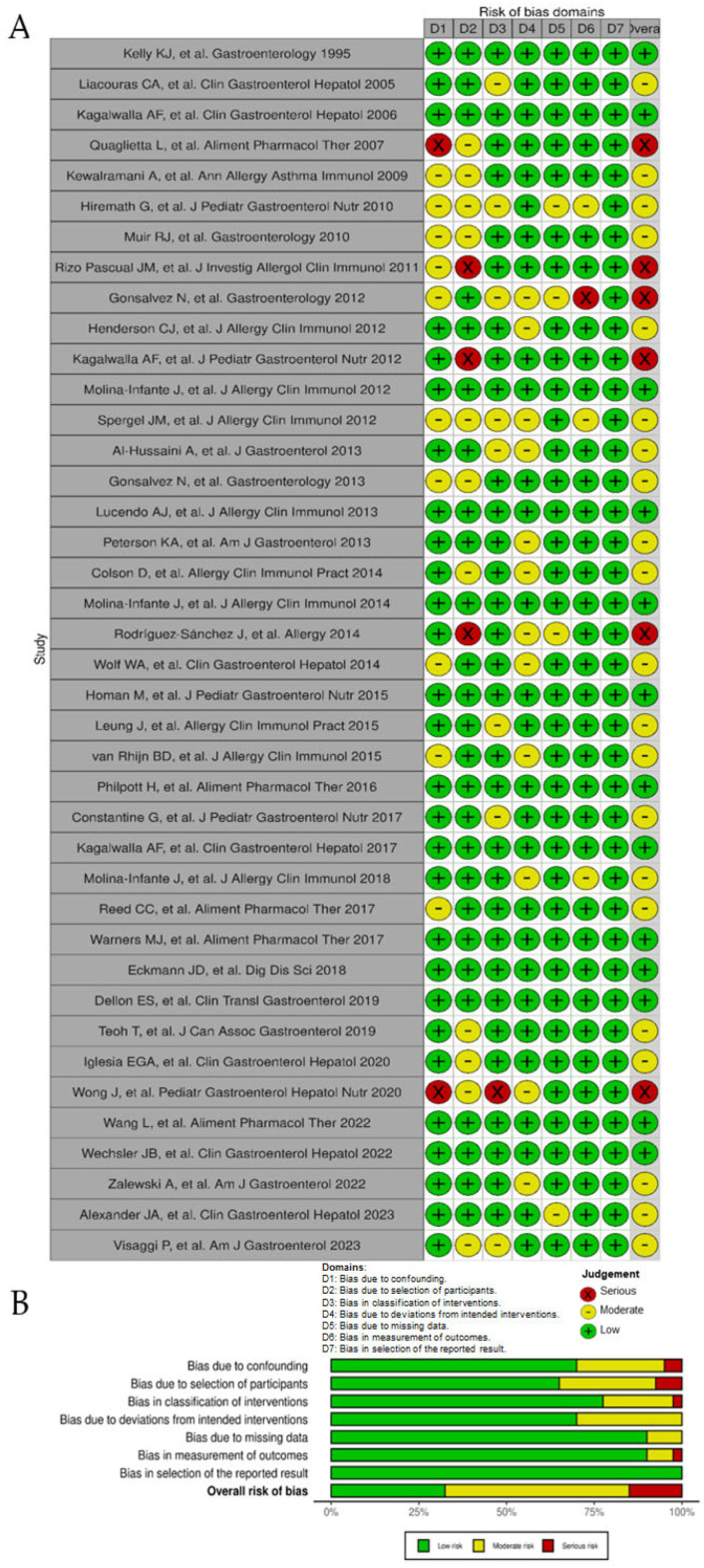
Risk of bias of observational studies included in the systematic review according to the Cochrane ROBINS-I tool. (**A**), ‘Traffic light’ plots of the domain-level judgments for each individual result [6,7,8,9,11,23,24,25,26,27,28,29,30,31,32,33,34,35,36,37,38,39,40,41,42,43,44,45,46,47,48,49,51,52,53,54,55,56,57,58]. (**B**), Weighted bar plots of the distribution of risk of bias judgements within each bias domain.

**Figure 3:**
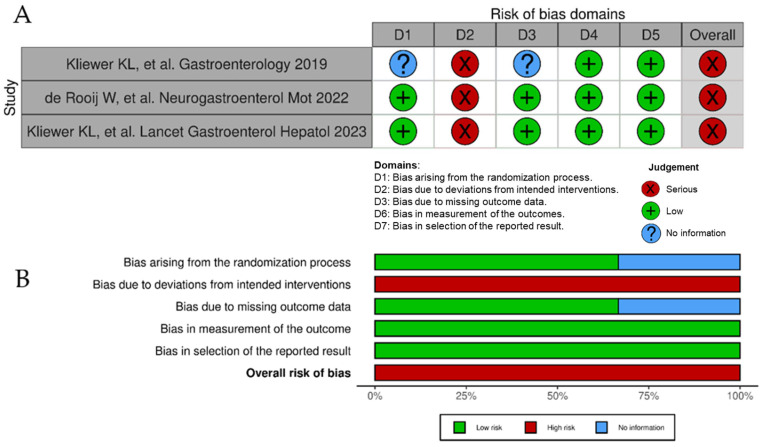
Risk of bias of randomized controlled trials included in the systematic review according to the Cochrane RoB 2 tool. (**A**), ‘Traffic light’ plots of the domain-level judgments for each individual result [12,50,59]. (**B**), Weighted bar plots of the distribution of risk of bias judgements within each bias domain.

Among all 40 observational studies included, only 13 studies were judged as low risk of bias, 21 had a moderate risk of bias [due to raised concerns on some specific items], whereas 6 presented a high or very high risk of bias (Figure 2A). Poor control of potential confounding factors, patient selection bias, and risk for deviation from intended dietary interventions were the main domains for risk of bias (Figure 2B). As for the three randomized controlled trials, their results were all considered of risk high of bias (Figure 3A) due to serious concerns regarding deviations from the intended intervention (low adherence to the most restrictive dietary options) that were likely to have affected primary outcomes (Figure 3B). 

**Table 1:** Baseline characteristics of the Studies Included.

First Author, Publication Years	Design	Period	Country	N Patients (Complete Diet)	Population	Male/Female (%)	Dietary Treatment	Diet Duration (Weeks)	N Histologic Responders (%)	N. Clinical Responders (%)	Clinical Response Method
Kelly et al., 1995 [23]	Prospective	1992–1994	USA	10	Children/Adolescents (0–12.5)	60/40	Elemental	At least 6	9/10 (90%)	10/10 (100%)	Symptom improvement
Liacouras C et al., 2005 [24]	Retrospective	1994–2004	USA	239	Children	74.5/25.5	Allergy-test directed diet (SPT, APT)	4–6	18/75 (24%)	18/75 (24%)	Symptom improvement
							Elemental	4–6	160/164 # (97.6%)	160/164 # (97.6%)	Symptom improvement
Kagalwalla AF et al., 2006 [25]	Retrospective	2001–2005	USA	60	Children	80/20	SFED	6	26/35 (74.3%)	32/35 (91.4%)	Symptom improvement
							Elemental	6	22/25 (88%)	25/25 (100%)	Symptom improvement
Quaglietta et al., 2007 [26]	Prospective	2005–2006	Italy	7	Children	-	Allergy-test directed diet (SPT, APT)	24	0/7 (0%)	-	Symptom improvement
Kewalramani et al., 2009 [55]	Prospective	-	USA	13	Children/Adolescents (1–19)	-	Allergy-test directed diet (SPT, ImmunoCAP)	12	6/13 (46.1%)	-	-
Hiremath G et al., 2010 [56]	Retrospective	-	USA	13	Children	70/30	Allergy-test directed diet (SPT, APT)	-	8/13 (61.5%)	-	-
Muir RJ et al., 2010 [57]	Prospective	-	Australia	13	Children/Adolescents (1–15)	84.6/15.4	SFED	6	5/13 (38.5%)	13/13 (100%)	Symptom improvement
Rizo-Pascual JM et al., 2011 [27]	Prospective	2001–2009	Spain	17 (14)	Children (2.8–14.5)	82.4/17.6	Elemental	8	3/3 (100%)	3/3 (100%)	Asymptomatic
							Allergy-test directed diet (IgE, SPT)	8	5/11 (45.4%)	5/11 (45.4%)	Asymptomatic
Gonsalves N et al., 2012 [6]	Prospective	2006–2010	USA	50	Adolescents/Adults (19–76)	50/50	SFED	6	37/50 (74%)	47/50 (94%)	Dysphagia resolution
Henderson C et al., 2012 [28]	Retrospective	1999–2011	USA	95	Children/Adolescents (<21)	77.9/22.1	Elemental	12	47/49 (95.9%)	-	-
							SFED	12	21/26 (80.8%)		
							Allergy-test directed diet (SPT, APT)	12	15/23 (65.2%)		
Kagalwalla AF et al., 2012 [29]	Retrospective	2006–2011	USA	111	Children	-	Allergy-test directed diet	6	52/82 (63.4%)	-	Symptom improvement
							Elemental	6	10/12 (83.3%)	-	
							OFED (milk)	6	11/17 (64.7%)	-	
Molina Infante J et al., 2012 [30]	Prospective	-	Spain	22	Adolescents/Adults (>18)	77.3/22.7	Allergy-test directed diet (SPT, PPT, APT)	6	4/15 # (26.7%)	4/15 (26.7%)	Symptom improvement
Spergel J et al., 2012 [31]	Retrospective	2000–2011	USA	470	Children	-	Elemental	-	144/151 (95.4%)	- -	- -
							Allergy-test directed diet (IgE, SPT, APT)	-	169/319 (53%)		
Al-Hussaini A et al., 2013 [32]	Retrospective	2009–2012	Saudi Arabia	13	Children	61.5/38.5	Elemental	8	3/3 (100%)	-	-
							Allergy-test directed diet (SPT, RAST)	8	4/10 (40%)		
Gonsalves N et al., 2013 [58]	Prospective	-	USA	28	Both		FFED	6	15/28 (53.6%)	-	Symptom improvement
Lucendo AJ et al., 2013 [7]	Prospective	2008–2010	Spain	67	Adolescents/Adults (17–60)	82.1/17.9	SFED	6	49/67 (73.1%)	67/67 (100%)	Symptom improvement
Peterson et al., 2013 [33]	Prospective	2009–2011	USA	18	Adolescents/Adults (19–58)	55.6/44.4	Elemental	2–4	17/18 (94.4%)	-	Symptom improvement
Colson D et al., 2014 [34]	Retrospective	2006–2012	France	59	Children	62.7/37.8	SFED + AAF	8	35/51 (68.6%)	58/59 (98.3%)	Symptom improvement
							SFED	8	6/8 (75%)		
Molina-Infante J et al., 2014 [8]	Prospective	2012–2014	Spain	52	Adolescents/Adults (>14)	63.5/36.5	FFED	6	28/52 (53.8%)	35/52 (67.3%)	DSS
							SFED	6	34/47 (72.3%)		
Rodríguez-Sánchez J et al., 2014 [35]	Prospective	2011–2013	Spain	17	Adolescents/Adults (>14)	76.5/23.5	SFED	6	9/17 (52.9%)	-	VAS-EoE score
Wolf WA et al., 2014 [36]	Retrospective	2006–2012	USA	31	Adolescents/Adults (>18)	48.4/51.6	Allergy-test directed diet (SPT)	6	6/19 (31.6%)	15/22 (68.2%)	Symptom improvement
							SFED	6	5/9 (55.6%)	7/9 (77.8%)	
Homan M et al., 2015 [37]	Retrospective	2005–2012	Slovenia	25	Children/Adolescents (0–18)	92/8	SFED	-	8/13 (61.5%)	9/13 (69.2%)	Asymptomatic
							Allergy-test directed diet (IgE, SPT, APT)	-	9/19 (47.4%)	10/19 (52.6%)	
							Elemental	-	1/1 (100%)	-	
Leung J et al., 2015 [38]	Retrospective	-	USA	100 (34)	Children/Adolescents (8–18)	78/22	OFED (milk)	8	13/22 (59.1%)	-	-
							Elemental	8	12/12 (100%)		
van Rhijn B et al., J 2015 [39]	Prospective	-	Netherlands	15	Adults	86.7/13.3	Allergy-test directed diet (microarray)	6	1/15 (6.7%)	-	-
Philpott H et al., 2016 [40]	Prospective	2013–2015	Australia	56	Adolescents/Adults (>18)	67.9/32.1	SFED	At least 2	29/56 (51.8%)	-	-
Constantine G et al., 2017 [41]	Retrospective	2006–2012	USA	14	Adolescents/Children	85.7/14.3	Allergy-test directed diet (APT, SPT)	At least 12	6/10# (60%)	9/14 (64%)	Symptom improvement
Kagalwalla AF et al., 2017 [9]	Prospective	2011–2016	USA	78	Children/Adolescents (<18)	66.7/33.3	FFED	6–8	50/78 (64.1%)	-	
Molina-Infante J et al., 2017 [42]	Prospective	2014–2016	Spain	130	Both	72.3/27.7	TFED	6	56/130 (43.1%)	98/130 (75.4%)	DSS
							FFED	6	66/110 (60%)		
							SFED	6	74/93 (79.6%)		
Reed C et al., 2017 [43]	Retrospective	2008–2017	USA	52 (50)	Adolescents/Adults (>18)	59.6/40.4	SFED	-	8/18 (44.4%)	38/52 (73.1%)	Dysphagia resolution
							Allergy-test directed diet (SPT, RAST)	-	11/32 (34.4%)		
Warners MJ et al., 2017 [44]	Prospective	2014–2015	Netherlands	21 (17)	Adults	70.6/29.4	Elemental	4	12/17 (70.6%)	17/17 (100%)	SDI and RDQ
Eckmann JD et al., 2018 [45]	Prospective	2014–2016	USA	8	Adults	50/50	SFED *	6	7 (1 *)/8 (87.5%)	8/8 (100%)	MDQ-30
Dellon ES et al., 2019 [46]	Prospective	-	USA	43	Adolescents/Adults (>18)	-	SFED	6	15/24 (62.5%)	-	-
							Allergy-test directed diet (IgG4)	6	4/19 (21.1%)		
Kliewer K et al., 2019 [59]	Prospective RCT	-	USA	63	Children/Adolescents (6–17)	-	OFED (milk)	12	15/34 # (44.1%)	-	PEESS V2.0
							FFED	12	7/17 # (41.2%)		
Teoh T et al., 2019 [47]	Retrospective	2013–2016	Canada	31	Children/Adolescents (<16)	83.9/16.1	OFED (milk)	8	18/31 (58.1%)	28/31 (90.3%)	Symptom improvement
Iglesia E et al., 2020 [48]	Retrospective	-	USA	8	Adolescents/Adults (12–67)	22/78	TFED	6–8	2/2 (100%)	7/8 (78%)	-
							FFED	6–8	3/3 (100%)		
							SFED	6–8	6/8 (75%)		
Wong J et al., 2020 [49]	Retrospective	-	USA	152 (21)	Children/Adolescents (<21)	76.3/23.7	OFED (dairy)	-	3/12 (25%)	-	-
							SFED	-	5/9 (55.6%)		
de Rooij WE et al., 2022 [50]	Prospective RCT	2017–2020	Netherlands	41	Adults	64/40	FFED	6	5/20 (25%)	-	SDI
							FFED + AAF	6	10/21 (47.6%)		
Wang L et al., 2022 [51]	Retrospective	2012–2019	USA	68	Adolescents/Adults (>18)	52.9/47.1	SFED *	6	42 (4 *)/68 (55.9%)	-	-
Wechsler JB et al., 2022 [11]	Prospective	2012–2017	USA	41	Children/Adolescents (2–18)	75.6/24.4	OFED (milk)	8–12	21/41 (51.2%)	37/41 (90.2%)	-
Zalewski A et al., 2022 [52]	Retrospective	2006–2021	USA	213	Adolescents/Adults	54/46	SFED *	6	123 (8 *)/213 (57.7%)	164/213 (77%)	Symptom improvement
Alexander JA et al., 2023 [53]	Prospective	2016–2018	USA	40	Adolescents/Adults (18–65)	-	SFED *	6	30 (2 *)/40 (75%)	-	EEsAI PRO
Kliewer K et al., 2023 [12]	Prospective RCT	2016–2019	USA	129	Adolescents/Adults (>18)	54.3/45.7	OFED (milk)	6	23/67 (34.3%)	-	EEsAI PRO
							SFED	6	25/62 (40.3%)		
Visaggi P et al., 2023 [54]	Retrospective	2017–2022	Italy & UK	58	Adults (>18)		SFED	6	33/58 (56.9%)	21/28 (75%)	Symptoms improvement

RCT, randomized controlled trial; M, male; F, female; SFED, six-food elimination diet; FFED, four-food elimination diet; TFED, two-food elimination diet; OFED, one-food elimination diet; RAST, radioallergosorbent test; SPT, skin prick test; APT, atopy patch test; PPT, prick-prick tests; AAF, amino acid formula; EEsAI PRO, The Eosinophilic Esophagitis Symptom Activity Index with Patient Reported Outcomes; SDI, Straumann Dysphagia Instrument; MDQ-30, 30-Day Mayo Dysphagia Questionnaire; DSS, Dysphagia Symptom Score; VAS-EoE Score, Visual Analogue Scale for Eosinophilic Esophagitis; RDQ, Reflux Disease Questionnaire; PEESS V2.0, Pediatric Eosinophilic Esophagitis Symptom Score v2.0. *: patients with extended SFED. #: missing biopsies/dropouts.

### 3.3. Effectiveness of Dietary Interventions to Induce Histologic Remission of EoE

The overall effectiveness for histologic remission of EoE for any dietary intervention was 60.6% (95% CI 54.6–66.5%; I^2^ 90%) (Table 2) (low certainty of the evidence; Table S3). Effectiveness was significantly superior in pediatric patients compared to adults (63.4% vs. 54.1%, respectively; *p* = 0.02) and was slightly higher in cohorts including patients of all ages (70.8%). Effectiveness was significantly higher for retrospective studies compared to prospective studies (66.4% vs. 54.4%, respectively; *p* = 0.04).

The effectiveness of each individual dietary intervention was analyzed in a separate meta-analyses. No significant publication bias was found in the funnel plot analysis (Figure S1) and Egger’s bias tests (Table 2).

#### 3.3.1. Elemental Diet

**Figure 4:**
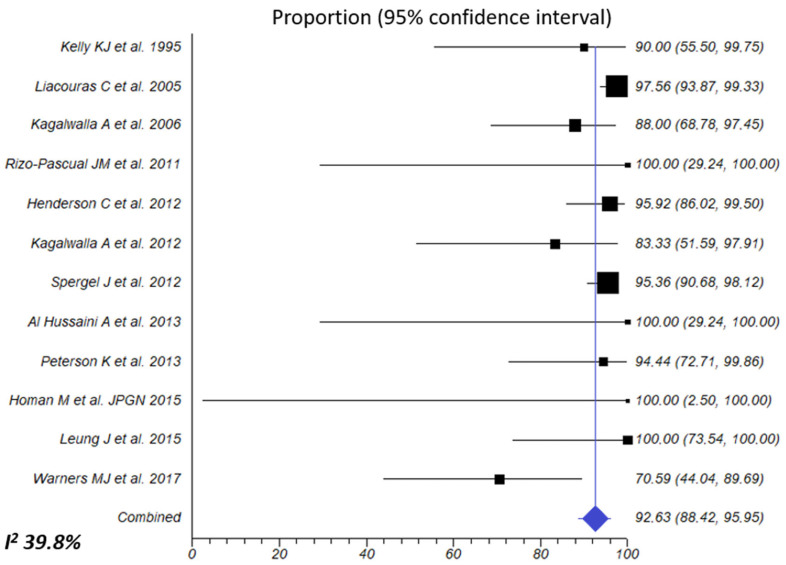
Overall combined effects of elemental diet for inducing histologic remission of EoE. Percentage of patients with <15 eos/HPF after dietary intervention was extracted from each article/abstract and 95% CIs were calculated using the exact binomial method. A random-effects model was used to calculate the overall effect size. The I^2^ of 39.8% indicates that intra-study differences (heterogeneity) account for only 39.8% of the variability in the overall effect size [23,24,25,27,28,29,31,32,33,37,38,44].

Exclusive feeding with an elemental diet was evaluated in 465 patients. The summary estimate for overall effectiveness was 94.5% (95% CI, 92.3–96.4%; I^2^ 39.8%), without observed publication bias (Table 2, Figure 4). Response was non-significantly superior in children compared to adults (95.2% vs. 82.7%; *p* = 0.210; moderate certainty of the evidence) and in retrospective compared to prospective studies (95.4% vs. 84.5%; *p* = 0.211).

#### 3.3.2. Empiric Six-Food Elimination Diet (SFED)

**Figure 5:**
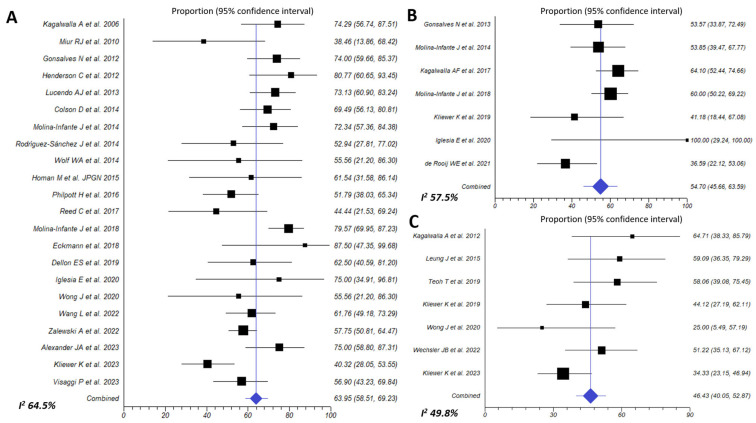
Efficacy of six-food elimination diet (**A**) [6,7,8,12,25,28,34,35,36,37,40,42,43,45,46,48,49,51,52,53,54,57], four-food elimination diet (**B**) [8,9,43,49,51,59,60] and one-food elimination diet (**C**) [11,12,29,38,47,49,59] in inducing histologic remission (<15 eos/HPF) in EoE patients.

The effectiveness of a SFED to induce histological remission of EoE was assessed in 22 studies gathering 995 patients. Effectiveness to induce histologic remission of EoE was 63.9% (95% CI, 58.5–69.2%; I^2^ 63.6%) (Figure 5A). Summary estimates of SFED effectiveness were extremely homogeneous when exclusive pediatric (67.5%), adult (63.5%) (high certainty of the evidence for both age groups), or multi-age cohorts (60.6%) were considered, and for prospective or retrospective study fashion (64.6% vs. 61.6%, respectively; *p* = 0.499) (Table 2).

#### 3.3.3. Empiric Four-Food Elimination Diet (FFED)

This dietary approach achieved a 54.7% (95% CI, 45.7–63.6%; I^2^ 57.7%) histological remission rate (<15 eos/HPF), when assessed in 7 prospective cohorts involving 329 EoE (Figure 5B). Effectiveness in children was 59.5% (high certainty of the evidence), no significantly superior to that observed in adults (52.7%; *p* = 0.423) (moderate certainty of the evidence) (Table 2).

#### 3.3.4. Empiric Two Food Elimination Diet (TFED)

Only two studies combining 132 patients overall have assessed this approach [3,48]. Histological remission rate was 44.3% (36.1–52.8%) (Table 2; Figure S2).

#### 3.3.5. One-Food Elimination Diet (OFED) (Milk Avoidance)

The simplest dietary approach to induce EoE remission was evaluated in 7 studies involving 224 patients, of whom 145 were children. Overall, 46.4% (95% CI, 40–52.9%; I^2^ 49.8%) patients achieved histological remission after avoidance of milk and dairy products from diet (high certainty of the evidence) (Figure 5C). Effectiveness trended to be superior in retrospective compared to prospective studies (54.6% vs. 41.6%; *p* = 0.309) (Table 2).

#### 3.3.6. Allergy Testing-Directed Food Elimination Diet

**Figure 6:**
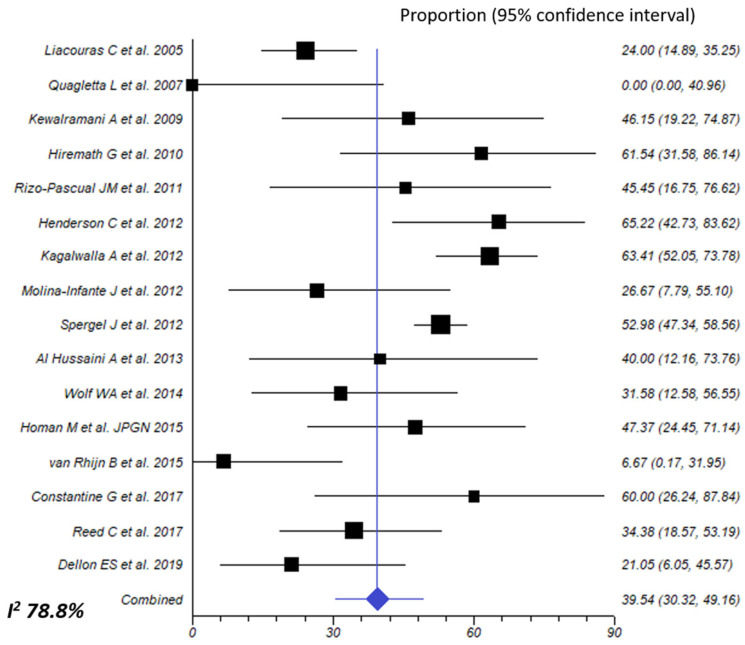
Overall effect size of allergy testing-directed food elimination for inducing histologic remission of eosinophilic esophagitis [24,26,27,28,29,30,31,32,36,37,39,41,43,46,55,56].

Elimination diet based on withdrawal of foods showing a positive result in either blood or skin allergy testing was assessed in 16 different cohorts, including 682 patients (572 children and 100 adults). Effectiveness to achieve histological remission was 39.5% (95% CI, 30.3–49.2%: I^2^ 78.8%) (Figure 6). A trend towards statistical significance was observed when comparing children to adults (45.7% vs. 26.4%, respectively; *p* = 0.063; very low of certainty evidence for both age groups), while there was a significant difference in favor of retrospective studies (47.5% vs. 24.2%; *p* = 0.012) (Table 2).

Summary estimates for effectiveness did not vary when different tests were compared, including a combination of skin prick test (SPT), atopy patch testing (APT), and serum food-specific IgE levels (Table 3).

### 3.4. Subgroup and Sensitivity Analyses 

The effectiveness of dietary interventions was slightly superior for most studies conducted in North America compared to those carried out in Europe, including elemental diet feeding (95.2% vs. 75.7%: *p* = 0.158), FFED (59.7% vs. 51.2%; *p* = 0.458), allergy testing-directed food elimination (45.5% vs. 25%; *p* = 0.055), and any dietary intervention (64.1% vs. 54%, respectively; *p* = 0.142). The only exception was a SFED, which trended to higher effectiveness in Europe over America (70% vs. 63.8%: *p* = 0.308).

As for sensitivity analyses, we calculated the effectiveness of the different dietary treatment approaches in studies with a low or moderate risk of bias, after excluding studies with a high risk of bias (Table 4). Summary estimates for overall effectiveness of any dietary intervention did not change significantly (63.7 [95%CI, 56.8–70.3]; I^2^ 90.8%), neither did results for SFEDs, FFEDs, nor OFEDs (values of 65.6%, 59.8%, and 55.2%, respectively). In addition, the effectiveness of allergy test-based food elimination maintained a similar value of 39.3% when studies with low or moderate risk of bias were exclusively considered.

### 3.5. Effectiveness of Dietary Interventions on Symptomatic Improvement of EoE

As for symptom assessment, only 29 studies out of the 43 included in this review evaluated the impact of the elimination diet on EoE symptoms. A simple acknowledgment of improvement without systematic evaluation was performed in 21 studies [6,7,23,24,25,26,27,29,30,33,34,36,37,41,43,47,52,54,57,58], non-validated symptom questionnaires were used in 5 studies [8,35,42,44,50], and 3 different validated questionnaires were used in the remaining 4 studies [12,45,53,59]. Histological remission generally led to symptomatic improvement or dysphagia resolution in most studies (Table 1). Due to heterogeneity and the lack of objective outcome measures for symptoms in the vast majority of included papers, we did not combine results for further analysis. 

## 4. Discussion

This updated meta-analysis proves that dietary therapy is an effective drug-free treatment for pediatric and adult EoE patients, with an overall histologic remission rate of 60%. In agreement with previous meta-analysis [4,60], an elemental diet remains the most effective strategy (92.6%), whereas allergy testing-guided elimination diets exhibit the lowest efficacy (39.5%). The present meta-analysis also confirms the increasing efficacy of empirical elimination diets with increasing levels of restriction, as demonstrated before by the 2-4-6 study [3]. These findings support the rationale of a step-up approach for dietary therapy in clinical practice. 

Noteworthy, the efficacy of any dietary therapy was significantly higher for children (compared to adults) and in retrospective studies (compared to prospective). Superiority in retrospective studies was observed for the elemental diet, OFED, and allergy testing-guided elimination diet, but not for the SFED. The majority of patients included in studies evaluating the aforementioned three dietary strategies were children, especially for the allergy testing-guided diet. Potential reasons for these findings may include better adherence in children thanks to the close surveillance provided by their parents and caregivers, more IgE-driven food polysensitization in children, and last but not least, a more common presence of one single food trigger, usually milk, in pediatric patients when compared with adults. A caveat to this novel observation is that the allergy testing-guided elimination diet (mostly in children) has been consistently defined here and before [4,60] as the dietary approach showing the highest variability and the highest risk of bias in literature. 

With the exception of the elemental diet, it is important to stress that all the remaining elimination diets suffered from variability in efficacy figures. This issue has been well described in previous meta-analyses, especially for allergy testing-based diets [4,60], which again showed the highest variability in the present manuscript. Nonetheless, variability in efficacy is novel for the SFED. In the first meta-analysis published in 2014 on dietary therapy for EoE, the efficacy of SFEDs was markedly homogeneous (72.8% and 71.3% for children and adults, respectively, I^2^ 0%) and thus widely generalizable [4]. In the present meta-analysis, the mean efficacy for SFEDs slightly decreased to 63.9%, with high variability (I^2^ 63.6%). Similar figures for SFEDs (efficacy 61.3%, I^2^ 73.5%) were reported in a recent meta-analysis on dietary therapy for EoE published in 2023 [60]. There might several reasons behind these conflicting and varying figures and trends in the recent literature for SFEDs. To begin with, 197 patients undertaking a SFED were included in the 2014 meta-analysis, of whom 85 (43%) were evaluated in Chicago, US, and 67 (34%) in Tomelloso, Spain [4]. In this updated meta-analysis, a 6- to 8-week SFED was the dietary scheme involving more patients (n = 995) worldwide, with most studies coming from multiple settings in the US and Spain, but also from France, Slovenia, Italy, the UK, and Australia. As such, heterogeneity in dietary information and dietitian follow-up (when available) in different centers may partially explain this conflicting trend. 

Counterintuitively, the aforementioned first randomized trial for SFEDs did not find differences in terms of histologic remission rate between a OFED and SFED (34% vs. 40%, *p* = 0.58) [12]. A histologic remission rate as low as 40% for SFED is one of the lowest efficacy figures ever reported (see Figure 5A) and is against data from almost all previous studies and meta-analyses [3,4,60]. In a second phase of this trial, non-responders to OFED were offered to escalate to a SFED. Given the fact that no differences were observed between both diets in the first analysis, no therapeutic gaining would have been expected for this step-up approach. However, 9 out 21 non-responders to a OFED (43%) were in histologic remission after escalation to a SFED, in line with all available evidence [3,4,60] and in disagreement with the initial results of the same trial. The most plausible reason for this contradiction is that patients were not fully adherent to the SFED in this first phase of the study, even though adherence to the SFED was reported to be as high as 97% in this trial. In fact, all three randomized controlled trials on dietary therapy included in this meta-analysis [12,50,59] were considered to have a high risk of bias due to concerns regarding low adherence to the most restrictive diets, likely underestimating the effectiveness of SFEDs and FFEDs. Actually, we do believe that poor adherence to highly restrictive diets is the major driver behind inconsistent data in the literature since we lack data on this issue in the vast majority of studies. 

Other factors contributing to decreasing or varying efficacy for SFEDs might include a shorter duration, differences in allowed foods during elimination diets, or the implementation of a diet during the pollen season. As for the duration of the diet, a recent meta-regression model observed that the duration of dietary therapy did not influence the effectiveness of dietary therapy [60]. In contrast, an Australian study demonstrated that extending the duration of a diet up to 13 more weeks was effective for a subset of non-responders to a 6-week empirical elimination diet [40]. In the present meta-analysis, a trend for higher efficacy with SFEDs in European studies (mostly Spanish) was shown, in comparison to those coming from the US. This discrepancy might be explained by the allowance of the consumption of non-wheat cereals and non-soy legumes in American studies [5,6,9,12]. A recent interesting study from Italy and the UK proved a lower response to a SFED in patients sensitized to pollens when adhering to a SFED during and outside of the pollen season were compared (21.4% vs. 77.3%; *p* = 0.003) [54]. Additionally, patients sensitized to pollen had significantly lower response to a SFED compared with those without sensitization (21.4% vs. 77.8%; *p* = 0.01). Collectively, these data point toward a decreased efficacy of SFEDs in either patients with pollen sensitization or diet implementation during the pollen season. Unfortunately, we lack data on this seasonal trend in most studies evaluating the efficacy of SFEDs, but no changes in effectiveness across seasons were found in previous large studies on FFEDs [8,9] or step-up empiric 2-4-6 food elimination diets [3], and season had no role in clinical presentation of EoE in a systematic review [61].

We herein first report the efficacy of a TFED (44%) in a meta-analysis, which comes from two studies including 132 patients (mostly adults) partaking in the same empirical TFED (milk and wheat) [3,48]. Milk and wheat have been consistently proven as the most common triggers for EoE in a recent meta-analysis on dietary therapy for EoE [60]. The efficacy of a TFED was slightly inferior to that for an OFED (46.4%), which is opposite to the belief that the higher the level of food restriction is, the greatest the efficacy. Since most patients adhering to a OFED were children, we suspect that similar efficacy for eliminating one or two foods might be explained by evaluation of each strategy in different age groups. We definitely need replication of these initial results for TFEDs in both children and adults in different settings to come to more solid conclusions about its efficacy. 

As for the OFED (milk elimination diet), its mean efficacy (47.8%) also showed variability when analyzed (I^2^ 49.8%), mostly in the pediatric population. Initial retrospective studies (2012–2019) showed higher efficacy figures from 58% to 64% [31,41,48,54], but histological remission rates are lower in more recent prospective studies or trials (2019–2023), ranging from 40% to 51% [11,12,59]. In prospective studies evaluating FFEDs and TFEDs from 2014 to 2018, milk-induced EoE (milk found as the only trigger after response to empirical diet and food reintroduction) was observed in 27% [8] and 19% [3] of adult patients, whereas higher numbers (56% [9] and 33% [3]) were reported in children. Aside from selection bias inherent to retrospective studies, another important caveat is that PPI therapy (despite the inclusion criteria for an OFED included >15 eos/HPF after PPI therapy) was maintained as a co-treatment with an OFED in most patients (>60%) in some pediatric studies [11,12,41,54]. In other studies, this information is not specifically provided [31,48,59]. Lack of data about clinical response to PPIs or the degree of histological response to previous PPIs (e.g., >15 eos/HPF but with a greater than 50–75% decrease in baseline esophageal eosinophilia after PPIs) casts the doubt on the possibility of including truly slow responders to PPI therapy within further considered “responders to OFED”, not to forget a potential synergistic effect of co-treatment with PPIs and an OFED. No trial has evaluated this hypothesis yet.

The strengths of the present meta-analysis comprise including a wide range of articles, regardless of the language; performing a thorough analysis of bias risk and first reporting a meticulous subgroup analysis with novel data; retrieving more studies and patient data than any other previous meta-analysis; and performing a rigorous assessment of certainty evidence of most important results according to GRADE. Unlike the most recent systematic review [60], our selection of documents excluded all of those studies that evaluated a dietary intervention in combination with drugs and those case series that were selected for having presented a favorable response to a dietary intervention. Limitations for the results reported here are inherent to methodological drawbacks in studies dealing with dietary therapy (inconsistent symptom data, variability in diet implementation [e.g., elimination of wheat vs. all gluten containing cereals, elimination of soy vs. all legumes, empirical elimination + elimination of foods exhibiting positive results in food allergy testing], different therapy durations, and lack of data on compliance with diet). We did not perform an analysis of food triggers resulting from studies including food reintroduction. Finally, the effect of dietary interventions on symptoms, when assessed, was done with variable methods, generally without the use of objective assessments, and, in the few cases where a measurement tool was used, it was a non-validated tool. All these reasons prevented us from combining the results in a meta-analysis. Although some validated and proprietary questionnaires for EoE symptoms exist, their use has been restricted to industry-sponsored drug trials due to their high cost to independent researchers. Future studies on diet therapy should address this issue.

## 5. Conclusions

This updated systematic review demonstrates that dietary therapy remains an effective and valuable therapeutic asset for pediatric and adult EoE patients. An elemental diet is the most effective approach, whereas empirical elimination diets are superior to allergy testing-guided diets. Our study reveal increasing efficacy with increasing levels of food restriction, confirming a step-up approach (starting with OFED/TFED) as the gold standard for clinical practice. We first report efficacy data for a TFED and discrepant effectiveness depending on age group, origin, and design of the study. Undoubtedly, these novel findings warrant further clarification.

## Figures and Tables

**Table 2:** Summary of histologic remission rates (<15 eos/HPF) and 95% CIs for the different dietary treatment options evaluated in children and adults. Subgroup analyses shown according to study design.

Dietary Treatment		N	Patients (n)	Overall Efficacy (%, 95% CI)	I^2^	Publication Bias ^†^
Any dietary treatment		66	2825	60.6% (54.6–66.5%)	90%	0.106
Exclusive feeding with an elemental diet		12	465	94.5% (92.3–96.4%)	39.8%	0.582
Six-food elimination diet		22	993	63.9% (58.5–69.2%)	63.6%	0.572
Four-food elimination diet		7	329	54.7% (45.7–63.6%)	57.5%	0.965 *
Two-food elimination diet		2	132	44.3% (36.1–52.8%) ^‡^	-	-
One-food (milk) elimination diet		7	224	46.4% (40–52.9%) ^‡^	49.8%	0.326 *
Allergy testing-directed food elimination		16	682	39.5% (30.3–49.2%)	78.8%	0.787
**Subgroups according to patients’ age**						
Any dietary treatment	Children	34	1389	63.4% (53.7–72.6%)	91.7%	0.328
	Adults	27	1104	54.1% (46.9–61.3%)	82.4%	0.482
	Both ages	9	332	70.8% (52.9–85.8%)	84.9%	0.728 *
Exclusive feeding with an elemental diet	Children	9	381	95.2% (92.9–97.1%) ^‡^	10.2%	0.324 *
	Adults	2	35	82.7% (69–93%) ^‡^	-	-
	Both ages	1	49	-	-	-
Six-food elimination diet	Children	5	137	67.5% (59.6–74.9%) ^‡^	34.7%	0.306 *
	Adults	14	600	63.5% (56.1–70.7%)	69.9%	0.518
	Both ages	4	256	60.6% (54.6–78%) ^‡^	51.8%	0.441 *
Four-food elimination diet	Children	4	129	59.5% (51–67.6%) ^‡^	0.2%	0.287 *
	Adults	4	197	52.7% (45.8–59.6%) ^‡^	55.3%	0.410 *
	Both ages	1	3	-	-	-
Two-food elimination diet	Children	1	25	-	-	-
	Adults	1	105	-	-	-
	Both ages	1	2	-	-	-
One-food (milk) elimination diet	Children	5	145	53.7% (45.7–61.6%)	0%	0.142 *
	Adults	1	67	-	-	-
	Both ages	1	12	-	-	-
Allergy testing-directed food elimination	Children	10	572	45.7% (34.4–57.2%)	79.6%	0.583 *
	Adults	5	100	26.4% (18.5–35.2%) ^‡^	21.9%	0.377 *
	Both ages	1	10	-	-	-
**Subgroup analysis according to study design**						
Any dietary treatment	Prospective	31	1203	54.4% (47.4–61.4%)	82.5%	0.590
	Retrospective	35	1622	66.4% (57.4–74.9%)	92%	0.182
Exclusive feeding with an elemental diet	Prospective	4	48	84.5% (73.5–93%) ^‡^	22.7%	0.768 *
	Retrospective	8	417	95.4% (93.2–97.2%) ^‡^	10.6%	0.340 *
Six-food elimination diet	Prospective	11	477	64.6% (55.2–73.5%)	76.3%	0.359
	Retrospective	11	516	61.6% (57.4–65.7%) ^‡^	24.3%	0.814
Four-food elimination diet	Prospective	6	326	55.4% (50.1–60.8%) ^‡^	53%	0.128 *
	Retrospective	1	3	-	-	-
Two-food elimination diet	Prospective	1	130	-	-	-
	Retrospective	1	2	-	-	-
One-food (milk) elimination diet	Prospective	3	142	41.6% (33.8–49.7%) ^‡^	35.4%	0.398 **
	Retrospective	4	82	54.6% (44–64.9%) ^‡^	42.5%	0.436 *
Allergy testing-directed food elimination	Prospective	6	80	24.2% (11.6–39.6%)	58.6%	0.031 *
	Retrospective	10	602	47.5% (37.5–57.6%)	76.5%	0.782 *

N, number of dietary interventions assessed overall; n, number of patients; I^2^, statistical inconsistence; ^†^, No asterisk means Begg–Mazumdar as bias indicator, * Egger bias indicator, ** Harbord bias indicator. ^‡^ Fixed effects.

**Table 3:** Summary estimates for the effectiveness of allergy testing-directed food elimination, according to the allergy test modalities used.

	N	Patients (n)	Overall Efficacy (%, 95% CI)	I^2^	Publication Bias *
SPT + APT	7	225	42.6% (24.4–61.2%)	86.3%	0.904
IgE + SPT	4	66	39.9% (28.8–51.6%)	0%	0.125
IgE + SPT + APT	2	338	52.7% (47.3–57.9%)	-	-

SPT, skin prick tests; APT, atopy patch testing; IgE, immunoglobulin E. N, number of dietary interventions assessed overall; n, number of patients; I^2^, statistical inconsistence. * Publication bias was assessed with Egger bias indicator.

**Table 4:** Subgroup analyses and sensitivity analysis comparing effectiveness of dietary therapy for eosinophilic esophagitis according to geographical origin of studies and risk of bias in source studies.

Dietary Treatment		N	Patients (n)	Overall Efficacy (%, 95% CI)	I^2^	Publication Bias ^†^
**Subgroups according to geographic origin of studies**						
Any dietary treatment	Europe	19	775	54% (44.9–63%)	82.3%	0.581
	North America	43	1968	64.1% (56.2–71.7%)	91.8%	0.320
Exclusive feeding with an elemental diet	Europe	3	21	75.7% (57–90.3%) ^‡^	0%	0.257 **
	North America	8	441	95.2% (93–97%) ^‡^	21.3%	0.074 *
Six-food elimination diet	Europe	7	354	70% (65.1–74.6%) ^‡^	49.7%	0.062 *
	North America	13	571	63.8% (56.4–70.9%)	61.6%	0.903
Four-food elimination diet	Europe	3	203	51.2% (38.2–64.1%)	69.6%	0.302 **
	North America	4	126	59.7% (51.2–68%) ^‡^	52.6%	0.883 *
Two-food elimination diet	Europe	1	-	-	-	-
	North America	1	-	-	-	-
One-food (milk) elimination diet	Europe	-	-	-	-	-
	North America	7	224	46.4% (40–52.9%) ^‡^	49.8%	0.326 *
Allergy testing-directed food elimination	Europe	5	67	25% (9.2–45.4%)	70.3%	0.131 *
	North America	10	605	45.5% (34.9–56.2%)	79.7%	0.624 *
**Subgroups according to risk of bias of source studies**						
Any dietary treatment	Low/moderate	51	2384	63.7% (56.8–70.3%)	90.8%	0.245
	High	15	441	49.4% (39.4–49.5%)	75.7%	0.559
Exclusive feeding with an elemental diet	Low/moderate	10	450	94.8% (92.6–96.6%) ^‡^	43%	0.152
	High	2	15	84.3% (63.9–97.2%)	-	-
Six-food elimination diet	Low/moderate	18	855	65.6% (60.2–70.9%)	57%	0.791
	High	4	138	55.8% (36.9–73.8%)	77.2%	0.943
Four-food elimination diet	Low/moderate	5	271	59.8% (54–65.5%) ^‡^	15.3%	0.410 *
	High	2	58	38.3% (26.5–50.9%) ^‡^	-	-
Two-food elimination diet	Low/moderate	2	132	44.3% (36.1–52.8%) ^‡^	-	-
One-food (milk) elimination diet	Low/moderate	3	94	55.2% (45.2–64.9%) ^‡^	0%	0.310 **
	High	4	130	40.1% (32–48.6%) ^‡^	52.8%	0.620 *
Allergy testing-directed food elimination	Low/moderate	13	582	39.3% (29.6–49.49%)	76%	0.392
	High	3	100	35.8% (5.8–74.3%)	87.8%	0.266 **

N, number of dietary interventions assessed overall; n, number of patients; I^2^, statistical inconsistence; ^‡^ Fixed effects; ^†^, no asterisk means Begg–Mazumdar bias indicator, * Egger bias indicator, ** Harbord bias indicator.

## Supplementary Materials

The following supporting information can be downloaded at: https://www.mdpi.com/article/10.3390/nu16142231/s1, Table S1. Search strategies using three bibliographic databases for documents that report on the effectiveness of dietary interventions to induce remission of eosinophilic esophagitis in patients of all ages. Table S2. Excluded studies after full-text review and reason for exclusion. Table S3. GRADE assessment for the effectiveness of the different dietary treatment approaches to induce histologic remission of eosinophilic esophagitis in patients of different age groups. Figure S1. Funnel plots of the studies reporting on the effectiveness of dietary interventions to induce remission of eosinophilic esophagitis considering any dietary intervention (A), an exclusive elemental diet (B), allergy-testing directed food elimination (C), a six-food elimination diet (D), a four-food elimination diet (E) and a one-food elimination diet (F). Figure S2. Summary estimates for the effectiveness of a two-food elimination diet.
