# Supplementary material for: Efficacy of Dietary Therapy for Eosinophilic Esophagitis in Children and Adults: An Updated Systematic Review and Meta-Analysis
Source: Nutrients. 2024 Jul 11;16(14):2231. doi: 10.3390/nu16142231 (PMC11279983; doi:10.3390/nu16142231)

nutrients

MDPI

Supplementary Material

Efficacy of Dietary Therapy for Eosinophilic Esophagitis in Children and Adults: An updated Systematic Review and Meta-analysis

Table S1. Search strategies carried out in three bibliographic databases for documents that report on the effectiveness of dietary interventions to induce remission of eosinophilic esophagitis in patients of all ages.

Search strategy	Bibliographic database	n documents
"Eosinophilic Esophagitis"[MeSH] OR "Eosinophilic oesophagitis"[MeSH] AND (diet OR dieta* OR diete*)	PubMed	347
((TITLE-ABS-KEY ("Eosinophilic Esophagitis")) OR (TITLE-ABS-KEY ("Eosinophilic oesophagitis"))) AND (TITLE-ABS-KEY ("diet*")) AND (EXCLUDE (DOCTYPE , "review"))	Scopus	1081
('eosinophilic esophagitis'/exp OR 'eosinophilic esophagitis') AND ('diet'/exp OR 'dieta*' OR 'diete*') AND [embase]/lim	Embase	824

Table S2. Excluded studies after full paper revision and reason for exclusion.

**Reference**	**Reason for Exclusion**
Abramson L, Smeekens JM, Kulis MD, Dellon ES. Food-specific IgA levels in esophageal biopsies are not sufficiently high to predict food triggers in eosinophilic esophagitis. Immun Inflamm Dis. 2023 Sep;11(9):e1029. doi: 10.1002/iid3.1029.	A re-analysis of the biopsies obtained at baseline endoscopies of Dellon et al. Clin Translational Gastroenterol).
Agulló-García A, Cubero JL, Lezaun A, Boné J, Guallar I, Colás C. Clinical and anatomopathological features of eosinophilic oesophagitis in children and adults. Allergol Immunopathol (Madr). 2020 Nov-Dec;48(6):560-567. doi: 10.1016/j.aller.2020.03.009	Retrospective study of dietary therapy without specifying the type of diet carried out or its effectiveness
Ahlawat R, Parikh NS, Jhaveri A. Triple Diagnosis of Crohn's Disease, Celiac Disease, and Eosinophilic Esophagitis in a Child With Siderius-Hamel Syndrome. WMJ. 2019 Oct;118(3):140-142	Single case report of a patient with EoE and Crohn's disease treated with gluten-free diet, prednisone and methotrexate.
Ahsan SD, Kammermeier J, Vora R, Mutalib M. Distal oesophageal spasm secondary to eosinophilic oesophagitis in a child: response to diet therapy. Frontline Gastroenterol. 2020 Jan;11(1):75-77. doi: 10.1136/flgastro-2019-101236	Single case report, no details on baseline or final eosinophil count in esophageal biopsies
Akhtar Ali S, Mathalikunnel A, Bhardwaj V, Braskett M, Pitukcheewanont P. Nutritional hypophosphatemic rickets secondary to Neocate® use. Osteoporos Int. 2019 Sep;30(9):1887-1891. doi: 10.1007/s00198-019-04836-8	Case report of 2 children with EoE (one of them also suffering from severe neuromuscular disorder), refractory to a 6-food diet, but with histological remission after elemental diet.

lexander RG, Ravi K, Collins MH, Lavey CJ, Snyder DL, Lennon RJ, Kassmeyer BA, Katzka DA, Alexander JA. Eosinophilic Esophagitis Histologic Scoring System: Correlation with Histologic, Endoscopic, and Symptomatic Disease and Clinical Use. Dig Dis Sci. 2023 Sep;68(9):3573-3583. doi: 10.1007/s10620-023-08029-6	Same patients used in a selected paper, in a paper focused on assessing relationships among symptoms, endoscopy and histology
Al-Hussaini A, Semaan T, El Hag I. Eosinophilic esophagitis in a developing country: is it different from developed countries? Gastroenterol Res Pract. 2013;2013:526037. doi: 10.1155/2013/526037	Post-hoc analysis of results of a previous trial, already selected for our systematic review
Arasi S, Costa S, Magazzù G, Ieni A, Crisafulli G, Caminiti L, Chiera F, Vaccaro M, Del Giudice MM, Pajno GB. Omalizumab therapy in a 13-year-old boy with severe persistent asthma and concomitant eosinophilic esophagitis. Ital J Pediatr. 2016 Mar 22;42:32. doi: 10.1186/s13052-016-0243-x	Single case report of a child with EoE not responding to both diets according to allergy and semi-elementary tests
Ari A, Morgenstern S, Chodick G, Matar M, Silbermintz A, Assa A, Mozer-Glassberg Y, Rinawi F, Nachmias-Friedler V, Shamir R, Zevit N. Oesophageal eosinophilia in children with coeliac disease. Arch Dis Child. 2017 Sep;102(9):825-829. doi: 10.1136/archdischild-2016-311944	A retrospective study of selected patients with EoE, celiac disease, and both conditions. Efficacy of gluten-free diet to resolve EoE was 22%
Armentia A, Martín-Armentia S, Martín-Armentia B, Santos-Fernández J, Álvarez R, Madrigal B, Fernández-González D, Gayoso S, Gayoso MJ. Is eosinophilic esophagitis an equivalent of pollen allergic asthma? Analysis of biopsies and therapy guided by component resolved diagnosis. Allergol Immunopathol (Madr). 2018 Mar-Apr;46(2):181-189. doi: 10.1016/j.aller.2017.11.001	An unrealistic description that following a high resolution IgE-guided food elimination diet and/or immunotherapy with pollen allergens cures EoE patients and celiacs.
Azzano P, Villard Truc F, Collardeau-Frachon S, Lachaux A. Children with eosinophilic esophagitis in real life: 10 years' experience with a focus on allergic management. Allergol Immunopathol (Madr). 2020 May-Jun;48(3):244-250. doi: 10.1016/j.aller.2019.07.013	The number of patients who improved their symptoms or histology with each of the therapies used (consisting of PPIs, topical or systemic corticosteroids, and various dietary interventions) is not mentioned.
Bianchi A, Calvani M, Leonardi L, Remotti D, Paparella R. Is a strict cow's milk-free diet necessary to treat milk oral immunotherapy-related eosinophilic esophagitis? Acta Biomed. 2021 Jul 1;92(3):e2021033. doi: 10.23750/abm.v92i3.10050	Single case report of a child with milk oral immunotherapy-triggered EoE, who presented remission after milk withdrawal, and tolerated cooked milk.
Bora V, Olive A, Chiou E, Raj P, Mehta D. Eosinophilic esophagitis with and without airway involvement in children - A comparative analysis. Int J Pediatr Otorhinolaryngol. 2020 Dec;139:110422. doi: 10.1016/j.ijporl.2020.110422	A description of the frequency different treatment strategies are used in patients with EoE that do and do not have airway symptoms; the effectiveness of each therapy is not reported.
Çakır M, Sağ E, Mungan S, Akbulut UE, Orhan F. Esophageal eosinophilia in children: A 6-year single-center experience. Turk J Pediatr. 2017;59(4):369-378. doi: 10.24953/turkjped.2017.04.002	A retrospective series of 5 patients with EoE who received dietary therapy as monotherapy. The effectiveness of any intervention was not reported.
Carlson DA, Hirano I, Zalewski A, Gonsalves N, Lin Z, Pandolfino JE. Improvement in Esophageal Distensibility in Response to Medical and	Six patients in this series used elimination diet (they do not mention which diet), in 3 patients together with PPI.

Diet Therapy in Eosinophilic Esophagitis. Clin Transl Gastroenterol. 2017 Oct 5;8(10):e119. doi: 10.1038/ctg.2017.47	The effectiveness of the diet in terms of resolution is not reported.
Chang N, Raja S, Betancourt R, Randall C, Keene S, Lilly A, Fowler M, Woosley JT, Shaheen NJ, Dellon ES. Generic Measures of Quality of Life Are Not Correlated with Disease Activity in Eosinophilic Esophagitis. Dig Dis Sci. 2021 Oct;66(10):3312-3321. doi: 10.1007/s10620-020-06719-z	An analysis of the impact of being on an elimination diet on quality of life, but not the effectiveness of the diet on inducing remission of EoE
Chawla K, Alabbas B, Sheth D, Papademetriou M. As Easy as EoE: A Novel and Effective Multidisciplinary Approach to Care of Patients with Eosinophilic Esophagitis in the Age of Biologics. Dig Dis Sci. 2020 Aug;65(8):2196-2202. doi: 10.1007/s10620-020-06366-4	An individual case report refractory to other therapies, including diet, who responded to Dupilumab.
Chen M, Ko HM, Riffle ME, Andreae DA, Cunningham-Rundles C, Chehade M, Maglione PJ. Eosinophilic esophagitis diagnosed in a patient with common variable immunodeficiency. J Allergy Clin Immunol Pract. 2016 Sep-Oct;4(5):995-7. doi: 10.1016/j.jaip.2016.03.023	Single case report of a patients with EoE and common variable immunodeficiency, who improved clinically with empirical SFED diet, but not histologically.
Collins CA, Palmquist J, Proudfoot JA, Qian A, Wangberg H, Khosh-Hemmat E, Dohil R, Aceves SS. Evaluation of long-term course in children with eosinophilic esophagitis reveals distinct histologic patterns and clinical characteristics. J Allergy Clin Immunol. 2019 Oct;144(4):1050-1057.e5. doi: 10.1016/j.jaci.2019.06.015	Prospective registry between 2011 and 2015 of 146 children with EoE treated with several therapies. Data on the effectiveness of each therapy are not provided, nor diet or the type of diet.
Crisafulli G, Caminiti L, Chiera F, Arasi S, Salzano G, Panasiti I, Barbalace A, Pajno GB. Omalizumab in children with severe allergic disease: a case series. Ital J Pediatr. 2019 Jan 14;45(1):13. doi: 10.1186/s13052-019-0602-5	A series of 8 allergic patients treated with Omalizumab, one of whom also had concomitant EoE. He responded to a semi-elemental diet, but abandoned it due to poor palatability.
de Rooij WE, Diks MAP, Warners MJ, Ampting MTJV, van Esch BCAM, Bredenoord AJ. Gene expression and clinical outcomes after dietary treatment for eosinophilic esophagitis: a prospective study. Neurogastroenterol Motil. 2022 Oct;34(10):e14367. doi: 10.1111/nmo.14367	A sub-analysis of biopsies taken from patients included in an study already selected for this review
Domenech Witek J, Gonzalez Mendiola R, Jover Cerdá V, Pereira González J, Carballas Vázquez C, Villas Martínez F, Rodríguez Pacheco R. Description of allergic phenotype in patients with eosinophilic oesophagitis: management protocol proposal. Sci Rep. 2023 Feb 8;13(1):2226. doi: 10.1038/s41598-023-29602-z	An allergen sensitization study in patients with EoE; no dietary therapy was developed after its results.
Doménech Witek J, Jover Cerdà V, Gil Guillén V, Doménech Clar JB, Rodríguez Pacheco R. Assessing eosinophilic cationic protein as a biomarker for monitoring patients with eosinophilic esophagitis treated with specific exclusion diets. World Allergy Organ J. 2017 Mar 23;10(1):12. doi: 10.1186/s40413-017-0143-6	Endoscopy with biopsies after the elimination diet based on allergy testing was not performed. Only clinical evaluation was done.
Erdle SC, Soller L, Avinashi V, Roberts H, Hsu E, Chan ES. Multiple shifting phenotypes with cow's milk: From eosinophilic esophagitis to	A case report description on a girl with EoE related to oral immunotherapy with cow's milk.

immediate hypersensitivity and back again. J Allergy Clin Immunol Pract. 2020 Mar;8(3):1117-1118. doi: 10.1016/j.jaip.2019.09.006	
Erwin EA, Kruszewski PG, Russo JM, Schuyler AJ, Platts-Mills TA. IgE antibodies and response to cow's milk elimination diet in pediatric eosinophilic esophagitis. J Allergy Clin Immunol. 2016 Aug;138(2):625-628.e2. doi: 10.1016/j.jaci.2016.01.048	A post hoc analysis of patients from another study; patients were treated simultaneously with PPI and milk exclusion diet.
Gómez Torrijos E, Moreno Lozano L, Extremera Ortega AM, González Jimenez OM, Mur Gimeno P, Borja Segade JM, Alfaya Arias T, García Rodríguez R. Eosinophilic Esophagitis: Personalized Treatment With an Elimination Diet Based on IgE Levels in Children Aged <16 Years. J Investig Allergol Clin Immunol. 2019 Apr;29(2):155-157. doi: 10.18176/jiaci.0358	Assessment of the effectiveness of an elimination diet based on serum IgE results against foods at very low levels (≥0.1 kU/L), which cannot be considered allergy-testing based, but close to empirical elimination diet, making it difficult to classify which strategy the authors follow.
Gomez Torrijos E, Rodriguez Sanchez J, Mendez Díaz Yesica C, Borja Segade JM, Galindo Bonilla PA, Feo-Brito JF, Garcia Rodríguez R. Eosinophilic Esophagitis: A New Possible Comorbidity in Difficult-to-Control Asthma? J Investig Allergol Clin Immunol. 2016;26(2):139-41. doi: 10.18176/jiaci.0034	A case report of a female patient with EoE who responded to SFED, but recurred after milk reintroduction.
González-Cervera J, Arias Á, Navarro P, Juárez-Tosina R, Cobo-Palacios M, Olalla JM, Angueira-Lapeña T, Lucendo AJ. Tolerance to sterilised cow's milk in patients with eosinophilic oesophagitis triggered by milk. Aliment Pharmacol Ther. 2022 Sep;56(6):957-967. doi: 10.1111/apt.17171	A study of tolerance to boiled milk in patients with EoE in whom milk was shown to trigger EoE.
Gottlieb SJ, Markowitz JE, Dellon ES. New IgE immediate hypersensitivity reactions on reintroduction of food restricted for treatment of eosinophilic esophagitis. Ann Allergy Asthma Immunol. 2019 Apr;122(4):419-420. doi: 10.1016/j.anai.2019.01.010	Two case reports on EoE patients who underwent an elimination diet and develop immediate reactions to milk after some months of avoidance.
Hamant L, Freeman C, Garg S, Wright BL, Schroeder S. Eosinophilic esophagitis may persist after discontinuation of oral immunotherapy. Ann Allergy Asthma Immunol. 2021 Mar;126(3):299-302. doi: 10.1016/j.anai.2020.12.007	Report of 4 cases of EoE supposedly induced after oral immunotherapy with foods, with variable results once the responsible foods were excluded.
Hill CA, Ramakrishna J, Fracchia MS, Sternberg D, Ojha S, Infusino S, Hartnick CJ. Prevalence of eosinophilic esophagitis in children with refractory aerodigestive symptoms. JAMA Otolaryngol Head Neck Surg. 2013 Sep;139(9):903-6. doi: 10.1001/jamaoto.2013.4171	Case series. After non-detailed medical treatment, EoE improved clinically by 50%; no results on endoscopic remission are provided.
Hirsch S, Cohen A, Rahbar R, Rubinstein E, Rosen R. Characterization of Eosinophilic Esophagitis in Infants and Toddlers. J Pediatr Gastroenterol Nutr. 2023 Jul 1;77(1):86-92. doi: 10.1097/MPG.0000000000003803	Small patients with EoE received any type of dietary treatment, most in combination with other drugs. The effectiveness or details of the dietary interventions carried out was not provided.
Ho HE, Chehade M. Development of IgE-mediated immediate hypersensitivity to a previously tolerated food following its avoidance	Four patients with EoE who achieved remission with FFED or SFED diets (which is not specified in each case) developed anaphylaxis to milk during its reintroduction.

for eosinophilic gastrointestinal diseases. J Allergy Clin Immunol Pract. 2018 Mar-Apr;6(2):649-650. doi: 10.1016/j.jaip.2017.08.014	
Hunter H, Pupinyte K, Wong T, Zeki S, Dunn JM, Toner E, Till SJ, Lomer MCE. Multidisciplinary approach to the management of adult eosinophilic oesophagitis in the United Kingdom. Clin Exp Allergy. 2018 Dec;48(12):1752-1756. doi: 10.1111/cea.13279	It was not possible to separate responder patients with <15 eos/HPF from those who reduce peak eosinophil counts over 50% from baseline.
Hunter H, Wong T, Winstanley A, Till SJ. Eosinophilic esophagitis linked to pollen food syndrome. J Allergy Clin Immunol Pract. 2018 Mar-Apr;6(2):667-668. doi: 10.1016/j.jaip.2017.06.040	A case report on a patient who achieved EoE remission after eliminating foods that cross-reacted with birch pollen.
Wechsler JB, Bolton SM, Amsden K, Wershil BK, Hirano I, Kagalwalla AF. Eosinophilic Esophagitis Reference Score Accurately Identifies Disease Activity and Treatment Effects in Children. Clin Gastroenterol Hepatol. 2018 Jul;16(7):1056-1063. doi: 10.1016/j.cgh.2017.12.019	The effectiveness of dietary therapy in pediatric EoE patients treated with some dietary options was not provided.
Johnson JB, Boynton KK, Peterson KA. Co-occurrence of eosinophilic esophagitis and potential/probable celiac disease in an adult cohort: a possible association with implications for clinical practice. Dis Esophagus. 2016 Nov;29(8):977-982. doi: 10.1111/dote.12419	Six patients selected by suffereing from PPI-refractory EoE associated to celiac disease followed a gluten free diet. After that 3 out of 5 who were re-evaluated with endoscopy normalized esophageal biopsies.
Jyonouchi S, Smith CL, Saretta F, Abraham V, Ruymann KR, Modayur-Chandramouleeswaran P, Wang ML, Spergel JM, Cianferoni A. Invariant natural killer T cells in children with eosinophilic esophagitis. Clin Exp Allergy. 2014 Jan;44(1):58-68. doi: 10.1111/cea.12201	Selection by convenience of 20 children with non-PPI responsive EoE; half of them became remission after different dietary strategies. No conclusion can be drawn about effectiveness of each dietary strategy to induce remission.
Kakiuchi T, Nakayama A, Matsuo M. Pediatric eosinophilic esophagitis effectively treated with a short-term 6-food-group elimination diet and reintroduction therapy: A case report. Medicine (Baltimore). 2019 Jun;98(26):e16243. doi: 10.1097/MD.0000000000016243	Single case report of a patient who responded to a SFED diet.
Ketchem CJ, Reed CC, Stefanadis Z, Dellon ES. Treatment with compounded fluticasone suspension improves the clinical, endoscopic, and histologic features of eosinophilic esophagitis. Dis Esophagus. 2021 Jul 12;34(7):doaa120. doi: 10.1093/dote/doaa120	Patients previously treated with diet or other corticosteroids are treated with fluticasone suspension. Of them, 43% had tried a food elimination diet (it does not say which one) and many had active disease. No conclusions can be drawn about the type of diet or its effectiveness, since they provide combined data of diet plus corticosteroids for primary or secondary lack of response.
Khakimova, R. F., Kamalova, A. A., Polyakov, N. S., Khomyakov, A. E., Nizamova, R. A., Zainetdinova, M. S., & Cheminava, L. D. Eosinophilic esophagitis in children: Experience in diagnosis, clinical observation in a multidisciplinary hospital. *Russian Journal of Allergy* 2023 *20*(1), 97-103. doi:10.36691/RJA2085	Case series of 7 EoE patients, I out of them treated with an elimination diet (not stated which one); due it was ineffective, the patient was switched to corticosteroids.

.

Kim JP, Weingart G, Hiramoto B, Gregory DL, Gonsalves N, Hirano I. Clinical outcomes of adults with eosinophilic esophagitis with severe stricture. Gastrointest Endosc. 2020 Jul;92(1):44-53. doi: 10.1016/j.gie.2020.01.015.	Retrospective analysis of EoE patients with severe strictures. Most patients were treated with more than one therapy at a time and many patients were exposed to different combinations of therapies at different times during follow-up.
Kovačić M, Unić J, Mišak Z, Jadrešin O, Konjik V, Kolaček S, Hojsak I. One-year outcomes in children with eosinophilic esophagitis. Esophagus. 2019 Apr;16(2):162-167. doi: 10.1007/s10388-018-0647-3.	Children with non-PPI responsive EoE were treated with diet according to allergy tests or SFED; data on effectiveness were not provided.
Kruszewski PG, Russo JM, Franciosi JP, Varni JW, Platts-Mills TA, Erwin EA. Prospective, comparative effectiveness trial of cow's milk elimination and swallowed fluticasone for pediatric eosinophilic esophagitis. Dis Esophagus. 2016 May;29(4):377-84. doi: 10.1111/dote.12339.	A prospective study in which pediatric patients are allocated to receive treatment with fluticasone+PPI or milk elimination diet+PPI. Two therapeutic options applied simultaneously are, therefore, assessed.
Laserna-Mendieta EJ, Casabona S, Savarino E, Perelló A, Pérez-Martínez I, Guagnozzi D, Barrio J, Guardiola A, Asensio T, de la Riva S, Ruiz-Ponce M, Rodríguez-Oballe JA, Santander C, Arias Á, Lucendo AJ; EUREOS EoE CONNECT research group. Efficacy of Therapy for Eosinophilic Esophagitis in Real-World Practice. Clin Gastroenterol Hepatol. 2020 Dec;18(13):2903-2911.e4. doi: 10.1016/j.cgh.2020.01.024.	Data on effectiveness of empirical elimination diets (1, 2, 4, or 6 foods) used in the 1st or 2nd line of treatment are provided, but not individually for each therapeutic option.
Laserna-Mendieta EJ, Navarro P, Casabona-Francés S, Savarino EV, Pérez-Martínez I, Guagnozzi D, Barrio J, Perello A, Guardiola-Arévalo A, Betoré-Glaria ME, Blas-Jhon L, Racca F, Krarup AL, Gutiérrez-Junquera C, Fernández-Fernández S, la Riva S, Naves JE, Carrión S, García-Morales N, Roales V, Rodríguez-Oballe JA, Dainese R, Rodríguez-Sánchez A, Masiques-Mas ML, Feo-Ortega S, Ghisa M, Maniero D, Suarez A, Llerena-Castro R, Gil-Simón P, de la Peña-Negro L, Granja-Navacerrada A, Alcedo J, Hurtado de Mendoza-Guena L, Pellegatta G, Pérez-Fernández MT, Santander C, Tamarit-Sebastián S, Arias Á, Lucendo AJ; EUREOS EoE CONNECT Research group. Differences between childhood- and adulthood-onset eosinophilic esophagitis: An analysis from the EoE connect registry. *Dig Liver Dis.* 2023 Mar;55(3):350-359. doi: 10.1016/j.dld.2022.09.020.	The use of empirical food elimination diets is mentioned, as well as the overall effectiveness. The type of specific diet used is not detailed, however.
Leigh LY, Spergel JM. An in-depth characterization of a large cohort of adult patients with eosinophilic esophagitis. Ann Allergy Asthma Immunol. 2019 Jan;122(1):65-72.e1. doi: 10.1016/j.anai.2018.09.452.	The majority of patients who used dietary treatment did so together with medications, and the effectiveness of the diet used exclusively was not specified.
Letner D, Farris A, Khalili H, Garber J. Pollen-food allergy syndrome is a common allergic comorbidity in adults with eosinophilic esophagitis. Dis Esophagus. 2018 Feb 1;31(2). doi: 10.1093/dote/dox122.	Any dietary approach was followed by 39 EoE patients and 56.4% of them achieved remission or were in the process of identifying food triggers at the time of the

	study. The effectiveness of each type of diet used was not detailed.
Lexmond WS, Neves JF, Nurko S, Olszak T, Exley MA, Blumberg RS, Fiebiger E. Involvement of the iNKT cell pathway is associated with early-onset eosinophilic esophagitis and response to allergen avoidance therapy. Am J Gastroenterol. 2014 May;109(5):646-57. doi: 10.1038/ajg.2014.12.	Details on the type of diet used in these patients was not provided.
Lucendo AJ, Arias Á, González-Cervera J, Mota-Huertas T, Yagüe-Compadre JL. Tolerance of a cow's milk-based hydrolyzed formula in patients with eosinophilic esophagitis triggered by milk. Allergy. 2013 Aug;68(8):1065-72. doi: 10.1111/all.12200. .	Inclusion criteria for patients consisted in having responded to a dietary intervention, and milk identified as a trigger for EoE.
Lucendo AJ, Arias Á, González-Cervera J, Olalla JM, Molina-Infante J. Dual response to dietary/topical steroid and proton pump inhibitor therapy in adult patients with eosinophilic esophagitis. J Allergy Clin Immunol. 2016 Mar;137(3):931-4.e2. doi: 10.1016/j.jaci.2015.07.033.	A selection of non-consecutive patients, identified based on whether they responded to either diet or drugs, and subsequently, to the reverse alternatives.
Lyles JL, Martin LJ, Shoda T, Collins MH, Trimarchi MP, He H, Kottyan LC, Mukkada VA, Rothenberg ME. Very early onset eosinophilic esophagitis is common, responds to standard therapy, and demonstrates enrichment for CAPN14 genetic variants. J Allergy Clin Immunol. 2021 Jan;147(1):244-254.e6. doi: 10.1016/j.jaci.2020.10.017.	It not detailed which type of dietary intervention young children with EoE responded.
McMurray JC, Clair BS, Spriet SW, Min SB, Brooks DI, Mikita CP. Outcomes of eosinophilic esophagitis in patients managed in a multidisciplinary clinic. Allergy Asthma Proc. 2022 Jan 1;43(1):78-84. doi: 10.2500/aap.2022.43.210102.	The elimination diet used to induce EoE remission was not detailed, despite its effectiveness in achieving remission was provided. Some patients underwent diet therapy together with topical corticosteroids.
Min S, Shoda T, Wen T, Rothenberg ME. Diagnostic merits of the Eosinophilic Esophagitis Diagnostic Panel from a single esophageal biopsy. J Allergy Clin Immunol. 2022 Feb;149(2):782-787.e1. doi: 10.1016/j.jaci.2021.07.032.	A collection of biopsies from patients from various centers in the USA; some patients were on dietary treatment. Type of diet or effectiveness was not specified.
Morales-Cabeza C, Infante S, Cabrera-Freitag P, Fuentes-Aparicio V, Zubeldia JM, Álvarez-Perea A. Oral Immunotherapy and Risk of Eosinophilic Esophagitis in Children: 15 Years' Experience. J Pediatr Gastroenterol Nutr. 2023 Jan 1;76(1):53-58. doi: 10.1097/MPG.0000000000003631	Retrospective review of oral food immunotherapy - induced EoE cases and final outcomes.
Morris DW, Stucke EM, Martin LJ, Abonia JP, Mukkada VA, Putnam PE, Rothenberg ME, Fulkerson PC. Eosinophil progenitor levels are increased in patients with active pediatric eosinophilic esophagitis. J Allergy Clin Immunol. 2016 Sep;138(3):915-918.e5. doi: 10.1016/j.jaci.2016.03.027.	Study on eosinophils progenitors in the blood of children with EoE, some of whom were receiving dietary treatment, either for induction or maintenance of disease remission. Type of diet and effectiveness are not provided.

Nistel M, Andrews R, Furuta GT, Atkins D. Elimination Diet or Swallowed Topical Steroid Treatment of Pediatric Eosinophilic Esophagitis: Five-Year Outcomes. J Allergy Clin Immunol Pract. 2023 Aug;11(8):2516-2523.e2. doi: 10.1016/j.jaip.2023.05.036.	Overall 16 children received dietary treatment for EoE, but half of them with concomitant PPI. The food elimination strategy was mostly empirical by consensus of the allergist, gastroenterologist and dietitian, but also according to positive SPT. The authors did not evaluate any predefined dietary scheme, but rather varied from one patient to another.
Nojkov B, Amin M, Ghaith G, Cappell MS. A Statistically Significant Association Between Esophageal Granular Cell Tumors and Eosinophilic Esophagitis: A 16-year Analysis at Two Large Hospitals of 167,434 EGDs. Dig Dis Sci. 2017 Dec;62(12):3517-3524. doi: 10.1007/s10620-017-4802-9. Epub 2017 Oct 24. PMID: 29064014.	A search of EoE cases over 16 years identified that 5 patients had associated granular cell tumors.
Oliva S, Dias JA, Rea F, Malamisura M, Espinheira MC, Papadopoulou A, Koutri E, Rossetti D, Orel R, Homan M, Bauraind O, Auth MK, Junquera CG, Vande Velde S, Kori M, Huysentruyt K, Urbonas V, Roma E, Fernández SF, Domínguez-Ortega G, Zifman E, Kafritsa P, Miele E, Zevit N; ESPGHAN EGID Working Group. Characterization of Eosinophilic Esophagitis From the European Pediatric Eosinophilic Esophagitis Registry (pEEr) of ESPGHAN. J Pediatr Gastroenterol Nutr. 2022 Sep 1;75(3):325-333. doi: 10.1097/MPG.0000000000003530.	Some pediatric patients with EoE not responding to PPI achieved remission after dietary therapy, without specifying which one.
Patton T, Chugh A, Padhye L, DeGeeter C, Guandalini S. Pediatric Celiac Disease and Eosinophilic Esophagitis: Outcome of Dietary Therapy. J Pediatr Gastroenterol Nutr. 2019 Aug;69(2):e43-e48. doi: 10.1097/MPG.0000000000002343.	Retrospective chart review to identify patients with concurrent EoE and celiac disease. It is an intentional selection of patients, therefore the effectiveness of the therapy may be biased by the selection.
Pehrsson M, de Rooij WE, Bay-Jensen AC, Karsdal MA, Mortensen JH, Bredenoord AJ. Extracellular matrix remodeling proteins as biomarkers for clinical assessment and treatment outcomes in eosinophilic esophagitis. BMC Gastroenterol. 2023 Oct 16;23(1):357. doi: 10.1186/s12876-023-02977-z. Erratum in: BMC Gastroenterol. 2023 Nov 22;23(1):407. doi: 10.1186/s12876-023-03048-z.	A subanalysis of a subgroup of patients from an already selected paper that does not provide additional data of interest.
Pesek RD, Rettiganti M, O'Brien E, Beckwith S, Daniel C, Luo C, Scurlock AM, Chandler P, Levy RA, Perry TT, Kennedy JL, Chervinskiy S, Vonlanthen M, Casteel H, Fiedorek SC, Gibbons T, Jones SM. Effects of allergen sensitization on response to therapy in children with eosinophilic esophagitis. Ann Allergy Asthma Immunol. 2017 Aug;119(2):177-183. doi: 10.1016/j.anai.2017.06.006.	Overall, 44.4% of EoE patients treated exclusively with diets (multiple options were possible) and 32 of them (44.4%) achieved remission. However, response rates to the different diatary alternatives were not analyzed separately. The authors did not differentiate whether remission was achieved with diet alone or associated with corticosteroids.

Philpott, H., & Dellon, E. Histologic improvement after 6 weeks of dietary elimination for eosinophilic esophagitis may be insufficient to determine efficacy. *Asia Pacific Allergy*, 2018; *8*(2). doi: 10.5415/apallergy.2018.8.e20	Some patients who no responded to a 6-week course of empirical elimination diet (several options) achieved remission after extending treatment length up to 13 weeks.
Reed CC, Safta AM, Qasem S, Angie Almond M, Dellon ES, Jensen ET. Combined and Alternating Topical Steroids and Food Elimination Diet for the Treatment of Eosinophilic Esophagitis. Dig Dis Sci. 2018 Sep;63(9):2381-2388. doi: 10.1007/s10620-018-4931-9.	Case series of 29 children treated with a 2-food diet combined with corticosteroids. Once remission is achieved, corticosteroids are discontinued and diet is maintained. EoE recurred in some of them.
Ridolo E, Martignago I, Pellicelli I, Incorvaia C. Assessing the Risk Factors for Refractory Eosinophilic Esophagitis in Children and Adults. Gastroenterol Res Pract. 2019 Jan 13;2019:1654543. doi: 10.1155/2019/1654543.	The authors differentiate between responders and non-responders to diet, but the number of patients who also received drug therapy is not shown.
Robson J, Laborda T, Fitzgerald S, Andersen J, Peterson K, O'Gorman M, Guthery S, Bennett-Murphy L. Avoidant/Restrictive Food Intake Disorder in Diet-treated Children With Eosinophilic Esophagitis. J Pediatr Gastroenterol Nutr. 2019 Jul;69(1):57-60. doi: 10.1097/MPG.0000000000002323.	Case reports of 2 children treated with drugs plus diet for EoE, who developed a food restriction disorder.
Rosen I, Mahamed A, Garah J, Magen-Rimon R, Shaoul R. The management and course of eosinophilic oesophagitis in Israeli children. Acta Paediatr. 2021 May;110(5):1653-1657. doi: 10.1111/apa.15746.	Case series or PPI-refractory EoE patients who underwent diatry therapy following 2 strategies: a) based on allergy testing ± milk exclusion, and b) SFED. In total, 23 patients achieved histological remission, but it is not clear with what type of diet.
Ruffner MA, Brown-Whitehorn TF, Verma R, Cianferoni A, Gober L, Shuker M, Muir AB, Liacouras CA, Spergel JM. Clinical tolerance in eosinophilic esophagitis. J Allergy Clin Immunol Pract. 2018 Mar-Apr;6(2):661-663. doi: 10.1016/j.jaip.2017.06.035.	Retrospective study of pediatric patients with PPI-refractory EoE selected by having achieve remission with a dietary intervention.
Hommeida S, Alsawas M, Murad MH, Katzka DA, Grothe RM, Absah I. The Association Between Celiac Disease and Eosinophilic Esophagitis: Mayo Experience and Meta-analysis of the Literature. J Pediatr Gastroenterol Nutr. 2017 Jul;65(1):58-63. doi: 10.1097/MPG.0000000000001499.	A 10-patient series of children presenting both EoE and celiac disease. In all of them, a gluten-free diet was established, and in addition, in some, corticosteroids or other additional dietary restrictions were given. Authors do not provide data on the effectiveness of the gluten-free diet in EoE.
Schupack DA, Ravi K, Geno DM, Pierce K, Mara K, Katzka DA, Alexander JA. Effect of Maintenance Therapy for Eosinophilic Esophagitis on Need for Recurrent Dilation. Dig Dis Sci. 2021 Feb;66(2):503-510. doi: 10.1007/s10620-020-06192-8.	A case series of EoE patients who underwent endoscopic dilatation at the beginning, in whom it was needed to dilate again over time was analyzed depending on whether or not they maintain remission with treatment. Some were on a diet, but they don't say which ones.
Shillitoe B, Lee JC, Hussien M, Beintaris I, Stothard M, Johnston M, Dallal HJ, Michaelis LJ, Attwood S, Dhar A. Clinical spectrum of paediatric and adult eosinophilic oesophagitis in the North East of	Three out or 45 patients achieved remission after elemental diet. However, there is no data about the total number of patients who were treated with an elemental diet.

England from 2016 to 2019. Frontline Gastroenterol. 2021 Jun 8;13(3):231-236. doi: 10.1136/flgastro-2021-101814.	
Sia T, Cunningham E, Miller M, Nitschelm R, Tanaka R, Epstein T, Garrett K, Huang A, Pak D, Scheve A, Leung J. Food elimination diet is a viable alternative therapy for eosinophilic esophagitis responsive to proton pump inhibitors. BMC Gastroenterol. 2023 Mar 9;23(1):60. doi: 10.1186/s12876-023-02703-9.	The effectiveness of diet therapy in patients with EoE responsive to PPI was assessed. The authors consider two cohorts: Retrospective and prospective. Effectiveness of diets in prospective cohort: 100% regardless of the option used. Unreliable results.
Sodikoff J, Hirano I. Proton pump inhibitor-responsive esophageal eosinophilia does not preclude food-responsive eosinophilic esophagitis. J Allergy Clin Immunol. 2016 Feb;137(2):631-3. doi: 10.1016/j.jaci.2015.07.008.	Case series of 5 patients selected for presenting a response to both PPI and dietary therapy independently.
Soller L, Mill C, Avinashi V, Teoh T, Chan ES. Development of anaphylactic cow's milk allergy following cow's milk elimination for eosinophilic esophagitis in a teenager. J Allergy Clin Immunol Pract. 2017 Sep-Oct;5(5):1413-1414. doi: 10.1016/j.jaip.2017.02.021.	Case report of a patient developing anaphylaxis due to milk after its elimination in a diet for the treatment of EoE.
Syrigou E, Angelakopoulou A, Zande M, Panagiotou I, Roma E, Pitsios C. Allergy-test-driven elimination diet is useful in children with eosinophilic esophagitis, regardless of the severity of symptoms. Pediatr Allergy Immunol 2015;26(4):323-9	Patients with EoE were artificially divided into two groups and treated with an elimination diet according to SPT + APT tests, together with corticosteroids. Once they achieved remission, the need for corticosteroids was reduced.
Terrados S, Villafana L, Antolín-Amérigo D, Camarero C, Martínez-Botas J, Sánchez-Ruano L, de la Hoz B. Effectiveness of allergy testing in milk induced eosinophilic esophagitis. Description and follow-up of patients. Allergol Immunopathol (Madr). 2020 Nov-Dec;48(6):576-581. doi: 10.1016/j.aller.2020.05.006.	Retrospective study of children with EoE treated with diet according to SPT results. In case of negative results, empirical or elemental diet was recommended. In 71% of patients, the causal food is identified, but it is not specified with what type of dietary intervention remission was achieved.
Topal E, Eğritaş O, Arga M, Sarı S, Poyraz A, Bakırtaş A, Dalgıç B. Eosinophilic esophagitis and anaphylaxis due to cow's milk in an infant. Turk J Pediatr. 2013 Mar-Apr;55(2):222-5.	Single case report of EoE along with anaphylaxis, both resolved after an exclusion diet.
Urganci N, Usta M, Civelek Z. Association of celiac disease with eosophageal eosinophilia and eosinophilic eosophagitis. Int J Clin Pract. 2021 Dec;75(12):e14836. doi: 10.1111/ijcp.14836. Epub 2021 Sep 22.	A large series of celiac children identified that 4 of them also had EoE. The effect of a gluten-free diet on esophageal biopsies was not reported.
Warners MJ, van Rhijn BD, Verheij J, Smout AJPM, Bredenoord AJ. Disease activity in eosinophilic esophagitis is associated with impaired esophageal barrier integrity. Am J Physiol Gastrointest Liver Physiol. 2017 Sep 1;313(3):G230-G238. doi: 10.1152/ajpgi.00058.2017.	Subanalysis of patients from an already selected study focused on analyzing epithelial permeability.
Warners MJ, Vlieg-Boerstra BJ, Verheij J, van Hamersveld PHP, van Rhijn BD, Van Ampting MTJ, Harthoorn LF, de Jonge WJ, Smout AJPM, Bredenoord AJ. Esophageal and Small Intestinal Mucosal Integrity in Eosinophilic Esophagitis and Response to an Elemental	Subanalysis of patients from an already selected study, focused on studying the integrity of the small intestine mucosa.

Diet. Am J Gastroenterol. 2017 Jul;112(7):1061-1071. doi: 10.1038/ajg.2017.107.	
Wenzel AA, Wadhwani N, Wechsler JB. Continued Basal Zone Expansion After Resolution of Eosinophilia in a Child With Eosinophilic Esophagitis on Benralizumab. J Pediatr Gastroenterol Nutr. 2022 Feb 1;74(2):e31-e34. doi: 10.1097/MPG.0000000000003319.	Case report of a girl with EoE refractory to PPI who achieved remission with diet.
Wichelmann TA, Hoff RT, Silas DN. Acute Herpes Simplex Esophagitis in an Immunocompetent Adult with Eosinophilic Esophagitis. Case Rep Gastroenterol. 2021 Dec 30;15(3):1003-1007. doi: 10.1159/000521124.	Case report of a patient with EGID with esophageal involvement, instead of EoE.
Wright BL, Kulis M, Guo R, Orgel KA, Wolf WA, Burks AW, Vickery BP, Dellon ES. Food-specific IgG4 is associated with eosinophilic esophagitis. J Allergy Clin Immunol. 2016 Oct;138(4):1190-1192.e3. doi: 10.1016/j.jaci.2016.02.024.	An assessment of the diagnostic value of IgG4 from esophageal samples of patients with known responses to dietary therapy.

EoE, Eosinophilic esophagitis; FFED, four-food elimination diet; SFED, six-food elimination diet; EGID, eosinophilic gastrointestinal disorder; IgG4, immunoglobulin G4; PPI, proton pump inhibitor; SPT, skin prick tests; APT, atopic patch testing

Table S3. GRADE assessment for the effectiveness of the different dietary treatment approaches to induce histologic remission of eosinophilic esophagitis in patients of different age groups.

Certainty assessment							№ of patients		Effect		Certainty	Importance
№ of studies	Study design	Risk of bias	Inconsistency	Indirectness	Imprecision	Other considerations	Dietary therapy	[Comparison]	Relative (95% CI)	Absolute (95% CI)		
**What is the overall effectiveness of dietary therapy to induce EoE remission in patients with EoE of all ages? (assessed with: Histologic remission rate [<15 eosinophils per HPF])**												
43	non-randomized studies	serious[a]	very serious[b]	not serious	not serious	strong association	1736/2825	0/0	not estimable		⊕⊕◯◯ Low	IMPORTANT
**What is the effectiveness of the exclusive elemental diet for EoE in children and adolescents? (assessed with: Histologic remission rate [<15 eosinophils per HPF])**												
9	non-randomized studies	serious[c]	not serious	not serious	not serious	very strong association	364/381 (95.5%)	0/0	not estimable		⊕⊕⊕◯ Moderate	IMPORTANT
**What is the effectiveness of the six-food elimination diet for EoE in adults? (assessed with: Histologic remission rate [<15 eosinophils per HPF])**												
14	non-randomized studies	not serious	serious[d]	not serious	not serious	strong association dose response gradient	384/602 (63.8%)	0/0	not estimable		⊕⊕⊕⊕ High	IMPORTANT
**What is the effectiveness of the six-food elimination diet for EoE in children and adolescents? (assessed with: Histologic remission [<15 eosinophils per HPF])**												
5	non-randomized studies	not serious	not serious	not serious	not serious	strong association dose response gradient	93/137 (67.9%)		not estimable		⊕⊕⊕⊕ High	IMPORTANT

Certainty assessment							№ of patients		Effect		Certainty	Importance
№ of studies	Study design	Risk of bias	Inconsistency	Indirectness	Imprecision	Other considerations	Dietary therapy	[Comparison]	Relative (95% CI)	Absolute (95% CI)		
**What is the effectiveness of the four-food elimination diet for EoE in children and adolescents? (assessed with: Histologic remission [<15 eosinophils per HPF[)**												
4	non-randomized studies	not serious	not serious	not serious	not serious	none	77/129 (59.7%)		not estimable		⨁⨁⨁⨁ High	CRITICAL
**What is the effectiveness of the four-food elimination diet for EoE in adults? (assessed with: Histologic remission [<15 eosinophils per HPF])**												
4	non-randomized studies	not serious	not serious	not serious	serious[e]	none	104/197 (52.8%)		not estimable		⨁⨁⨁◯ Moderate	CRITICAL
**What is the effectiveness of the one-food (milk) elimination diet for EoE in children and adolescents? (assessed with: Histologic remission [<15 eosinophils per HPF])**												
5	non-randomized studies	serious[f]	not serious	not serious	not serious	strong association all plausible residual confounding would reduce the demonstrated effect	78/145 (53.8%)		not estimable		⨁⨁⨁⨁ High	CRITICAL
**What is the effectiveness of allergy testing-directed food elimination diet for EoE in children and adolescents? (assessed with: Histologic remission [<15 eosinophils per HPF])**												
10	non-randomized studies	serious[g]	very serious[h]	very serious[i]	very serious[j]	strong association all plausible residual confounding would	286/572 (50.0%)		not estimable		⨁◯◯◯ Very low	IMPORTANT

Certainty assessment							№ of patients		Effect		Certainty	Importance
№ of studies	Study design	Risk of bias	Inconsistency	Indirectness	Imprecision	Other considerations	Dietary therapy	[Comparison]	Relative (95% CI)	Absolute (95% CI)		
						suggest spurious effect, while no effect was observed						
**What is the effectiveness of allergy testing-directed food elimination diet for EoE in adults? (assessed with: Histologic remission [<15 eosinophils per HPF])**												
5	non-randomized studies	not serious	very serious[k]	very serious[l]	not serious	all plausible residual confounding would reduce the demonstrated effect	26/100 (26.0%)		not estimable		⊕◯◯◯ Very low	IMPORTANT

CI: confidence interval

Explanations

a. Of a total of 43 studies, 21 presented a moderate risk of bias, 9 a high risk of bias and only 13 were considered low risk of bias.

b. Inconsistency measured by I^2 was 90%

c. Of the 10 studies, 2 had a high risk of bias, 5 were evaluated as having a moderate risk of bias, and the remaining 3 had a low risk of bias.

d. Inconsistency measured by I^2 is 69.9%

e. Inconsistency measured by I^2 is 55.3%

f. Out of the 5 studies, 2 were considered of high risk of bias, 2 of moderate risk of bias and only 1 of low risk of bias

g. Out of the 10 studies, 3 presented high risk of bias, 6 moderate risk of bias and only one a low risk of bias

h. The test to detect food sensitization to direct dietary avoidance varies wide among studies

i. Positive test results reflect prevalence of food sensitization in population. IgE mediated food allergy has not been clearly involved in pathophysiology of EoE.

j. Inconsistency measured by I^2 was 79.5%

k. Allergy test used to direct food avoidance varied from one study to the other

l. Positive test results reflect prevalence of food sensitization in population. IgE mediated food allergy has not been clearly involved in pathophysiology of EoE.

Figure S1. Funnel plots of the studies reporting on the effectiveness of dietary interventions to induce remission of eosinophilic esophagitis considering any dietary intervention (A), exclusive elemental diet (B), allergy-testing directed food elimination (C), six-food elimination diet (D), four-food elimination diet (E) and one-food elimination diet (F).

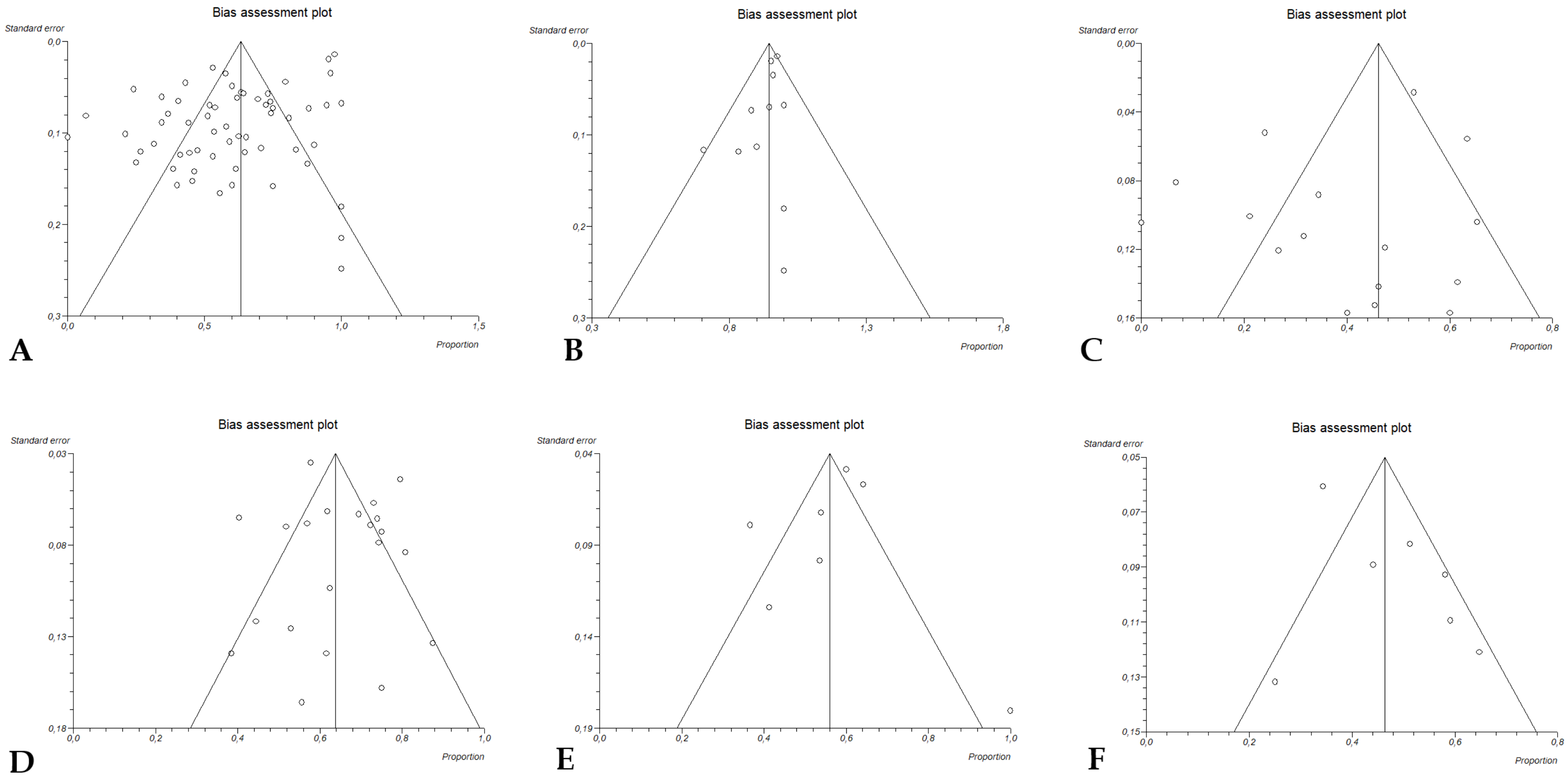

Figure S2. Summary estimates for the effectiveness of two-food elimination diet.

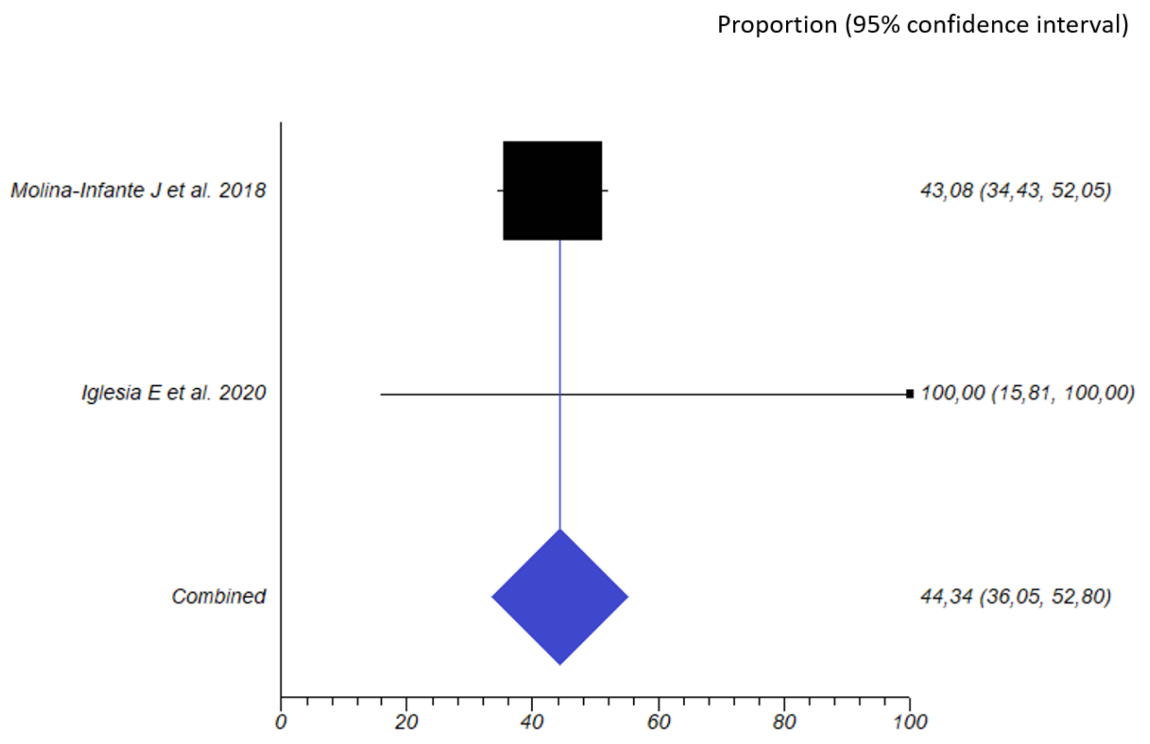

Supplement: Supplementary file 1 [file nutrients-16-02231-s001.zip › nutrients-3101756-supplementary.pdf]
